# Sociality sculpts similar patterns of molecular evolution in two independently evolved lineages of eusocial bees

**DOI:** 10.1038/s42003-021-01770-6

**Published:** 2021-02-26

**Authors:** Wyatt A. Shell, Michael A. Steffen, Hannah K. Pare, Arun S. Seetharam, Andrew J. Severin, Amy L. Toth, Sandra M. Rehan

**Affiliations:** 1 Department of Biology, York University, Toronto, ON Canada; 2 Department of Biological Sciences, University of New Hampshire, Durham, NH 03924 USA; 3 Office of Biotechnology, Iowa State University, Ames, IA 50011 USA; 4 Department of Entomology, Iowa State University, Ames, IA 50011 USA

**Keywords:** Social evolution, Evolutionary genetics

## Abstract

While it is well known that the genome can affect social behavior, recent models posit that social lifestyles can, in turn, influence genome evolution. Here, we perform the most phylogenetically comprehensive comparative analysis of 16 bee genomes to date: incorporating two published and four new carpenter bee genomes (Apidae: Xylocopinae) for a first-ever genomic comparison with a monophyletic clade containing solitary through advanced eusocial taxa. We find that eusocial lineages have undergone more gene family expansions, feature more signatures of positive selection, and have higher counts of taxonomically restricted genes than solitary and weakly social lineages. Transcriptomic data reveal that caste-affiliated genes are deeply-conserved; gene regulatory and functional elements are more closely tied to social phenotype than phylogenetic lineage; and regulatory complexity increases steadily with social complexity. Overall, our study provides robust empirical evidence that social evolution can act as a major and surprisingly consistent driver of macroevolutionary genomic change.

## Abstract (web summary)

Shell et al. compare the molecular evolution of bee genomes across species with varying social behaviours. Their findings indicate that eusocial lineages tend to have more gene family expansion than weakly social lineages. Transcriptomic data suggests that social phenotype is a consistent driver of macroevolutionary genomic change.

## Introduction

Sociogenomics has provided important advances in our understanding of the molecular basis of social life^1^. Studies have repeatedly shown that the evolution of animal sociality is strongly influenced and accompanied by a variety of genomic changes^2–5^. An emerging theme in sociobiology is the observation that, in addition to genes affecting social behavior, social life itself may drive new patterns and processes in genome evolution^6^. For example, it has been proposed that changes in demography, levels of selection, and the novel demands of social life can drive rapid sequence evolution, changes in genome organization, and other forms of genomic change. To date, however, there remains a paucity of genomic information for multiple closely-related species that vary in levels of sociality, leaving these ideas without robust empirical support.

Eusocial organisms demonstrate the most complex form of social organization in nature: a single reproductive queen is supported by hundreds or thousands of sterile offspring, all cooperatively working together to rear additional generations of brood^7,8^. Eusociality has evolved only rarely but has emerged more often among bees than in any other group (as many as four times^9–11^). As is demonstrated by the obligate social nesting of advanced eusocial bees, the emergence of social life marks a pivot point from individual to group living, making it one of the most consequential evolutionary transitions in the history of biological complexity on Earth^12^. The progression from solitary to eusocial life among the socially diverse bees thus presents unique opportunities to address how the transition to sociality—and a new level of biological organization (i.e. superorganismal)—may influence genome evolution.

It is generally thought that the evolutionary change from ancestral solitary life to group living in bees could not have occurred in a single, abrupt evolutionary step. Rather, evidence indicates that bees have collectively reverted to solitary life at least nine times following an emergence of group living^9–11^. Additionally, many extant bees demonstrate social forms that are not eusocial, such as subsocial (i.e. extended parental care) or incipiently social taxa (i.e. rudimentary but totipotent division of labor^13,14^). As synthesized by the social ladder framework^2^, the solitary ancestors of eusocial species thus likely underwent multifaceted, incremental, and largely reversible augmentations in social complexity, with relatively few lineages experiencing enough sustained selective pressure to cross a ‘point of no return’ into the obligate eusocial state^15–19^.

One core evolutionary-developmental theory states that, at its evolutionary origin, the obligate reproductive (queen) and non-reproductive (worker) caste system of advanced eusocial bees (e.g. *A. mellifera*) was necessarily underpinned by a decoupling of ancestrally maternal foraging/provisioning from egg-laying behaviors at the molecular level (i.e. ovarian groundplan hypothesis^14,18^). Studies among other group living and eusocial bees, however, suggest that social dynamics may emerge antecedent to the molecular division between reproductive and non-reproductive activity. For example, in many species of incipiently or facultatively social carpenter bees (Apidae: Xylocopinae), newly eclosed females do not immediately disperse, but instead “wait” in their natal nest to succeed the older nestmate as the dominant reproductive and forager^17,20^. Using Bayesian trait mapping analysis among 16 allodapine bee species (Xylocopinae: Allodapini) collectively demonstrating subsocial through advanced eusocial biology, Schwarz et al.^17^ found that this non-reproductive wait strategy was the most likely ancestral state for the group. This suggests that (i) evolutionary trajectories towards derived sociality may be highly lineage-specific and (ii) molecular decoupling of maternal pathways may not be necessary for quantifiable sociality to emerge.

To date, comparative sociogenomic studies among bees have largely focused on eusocial corbiculates (Apidae: Apinae) to make important foundational inferences into the factors contributing to the evolutionary emergence and elaboration of insect sociality^21,22^. Empirical insights from these works, however, are effectively limited to one particularly derived bee lineage. Moving forward, as genomic resources continue to be developed, the field of sociogenomics will benefit enormously by expanding into bee systems representative of other social lineages and phenotypes. This project is a step along that route, incorporating genome data from six carpenter bee species (Apidae: Xylocopinae), collectively representative of an independent origin of sociality, and a first-ever monophyletic dataset incorporating solitary (i.e. ancestral) through advanced eusocial (i.e. derived) taxa^11,17,23^. This unique dataset also affords us an opportunity to begin empirically discerning the degree to which the evolution of eusocial traits may have been driven by environmental constraints (i.e. adaptive change) versus shared ancestry (i.e. phylogenetic inertia^24,25^)—a largely open question within the field of social evolution. Though often handled separately, the effects of adaptive change and phylogenetic inertia are not mutually exclusive^24^; and comparative assessment of the molecular impact of each on the evolution of social traits would greatly inform further theoretical and empirical approaches.

Owing to their global distribution and rich social diversity, carpenter bees have long been the focus of illuminating phylogenetic and behavioral ecological research (e.g.^17,23,26–28^). To date, published genomic and transcriptomic resources for the Xylocopinae have been limited but highly informative resources for comparative studies of early social evolution^29–35^. Here we present four newly sequenced xylocopine genomes and transcriptomes and combine these with published genomic and transcriptomic data from 12 additional bee species to address three main questions. (1) Do genomes of independently evolved social bee lineages (Apinae and Xylocopinae) undergo parallel molecular changes during various stages of social evolution? As articulated by the social ladder framework^2^ and supported by previous comparative genomic research (e.g.^4,21,36^), we hypothesize that patterns of genome evolution (e.g. gene family expansions, rate of birth of novel genes, gene regulatory complexity) will be similar between lineages of comparable social evolutionary complexity despite phylogenetic distance. (2) How do rates of molecular evolution vary by social complexity and social lineage? We hypothesize, based on both theoretical^15^ and empirical support^37,38^, that rates of protein evolution will be(i) higher across socially derived lineages, and (ii) elevated among genes associated with caste roles^18^. (3) Are there regulatory elements associated with social traits that are conserved across evolutionary lineages of bees; and, do these elements allow for a disentangling of the influences of phylogenetic inertia from adaptive change on the emergence of social phenotypes? We hypothesize that while shared ancestry undoubtedly plays a role in the likelihood of social trait emergence *within* a given lineage, subsequent elaborations in the social form are likely attributable to environmental pressures acting consistently *across* lineages^18,24,25,39^. Covering 16 genomes and two well-represented and independent social lineages, this study represents the most comprehensive comparative analysis of social evolution in bees to date, and a “way forward” to investigate the tractability of the social ladder framework^2^. Further, this dataset provides an exciting opportunity to explore how adaptive change and phylogenetic inertia may influence the evolution of insect social complexity.

## Results and discussion

### Genome evolution

#### Sociality shapes gene family expansions and taxonomically restricted genes

The estimated genome sizes of our de novo assemblies (via SSpace, Trinity, and Gapfiller^40–42^) ranged from 280 Mb (*C. jap*onica) to 460 Mb (*E. robusta*) with final assembly N50s ranging from 54.9 kb (*C. japonica*) to 452 kb (*E. robusta*). These genomes are thus within the expected size range given previously published bee genome data (Data S1–S3). Analysis of Benchmarking Universal Single-Copy Orthologs (BUSCOs^43^) revealed that each assembly is highly complete, containing at least 95.9% complete Arthropod genes (Data S3; Fig. S5). A combination of RNA sequencing, de novo assembly and corrective editing via the MAKER2 pipeline^44^ yielded predicted gene counts consistent with previously published bee genomes (Data S2, S3).

**Fig. 1:**
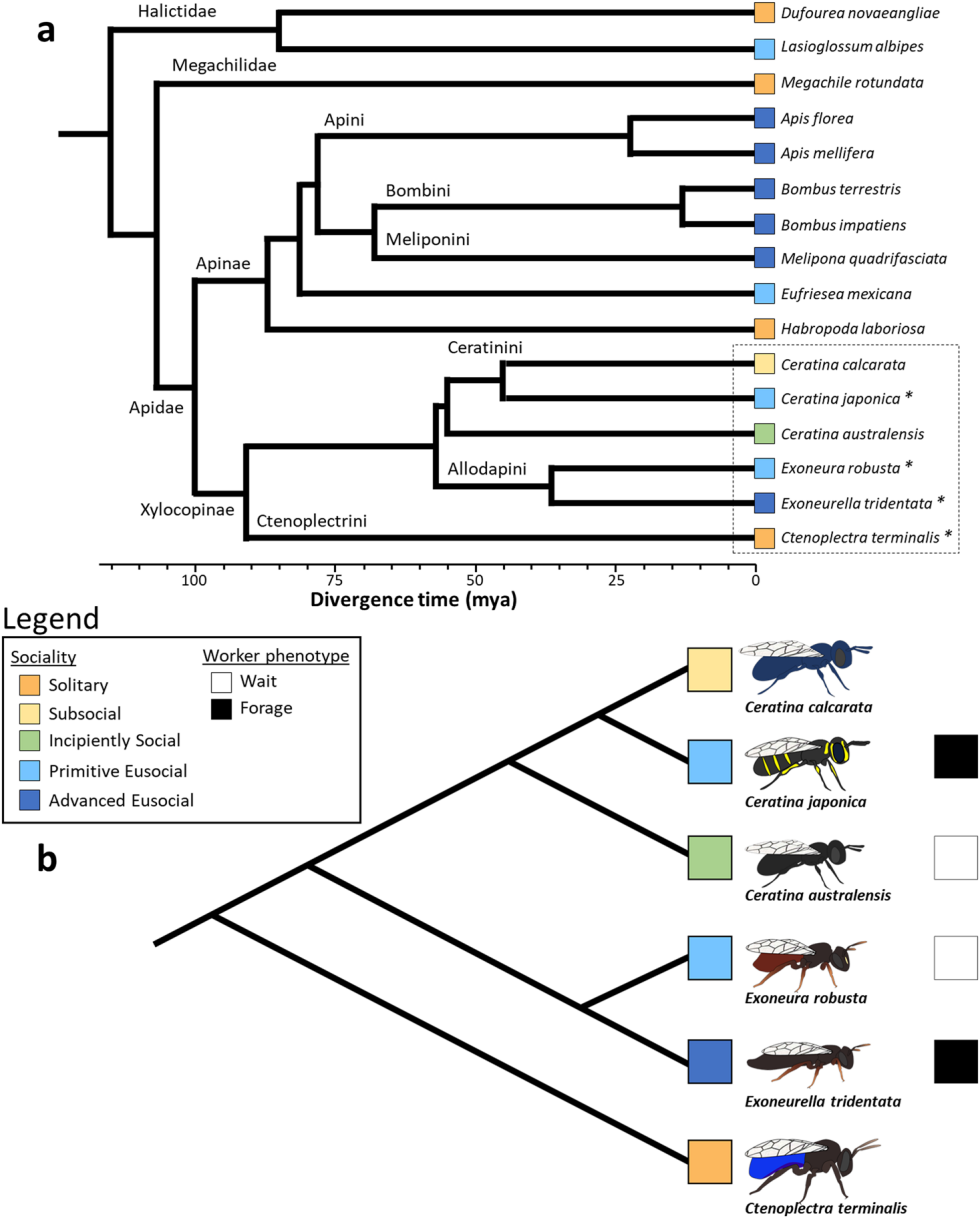
Sixteen bee comparative study phylogeny with trait mapping (Rehan et al.^11^; Bossert et al.^70^). **a** All species included in study with available genome data; asterisks identify species for which genome data were generated de novo during current study; hashed box identifies Xylocopine species which were also assessed via gene expression analyses. Divergence time in millions of years (mya) among lineages is provided. **b** Xylocopine species with social trait mapping. Lineage sociality and worker phenotypes are indicated in colored boxes as per legend.

**Fig. 2:**
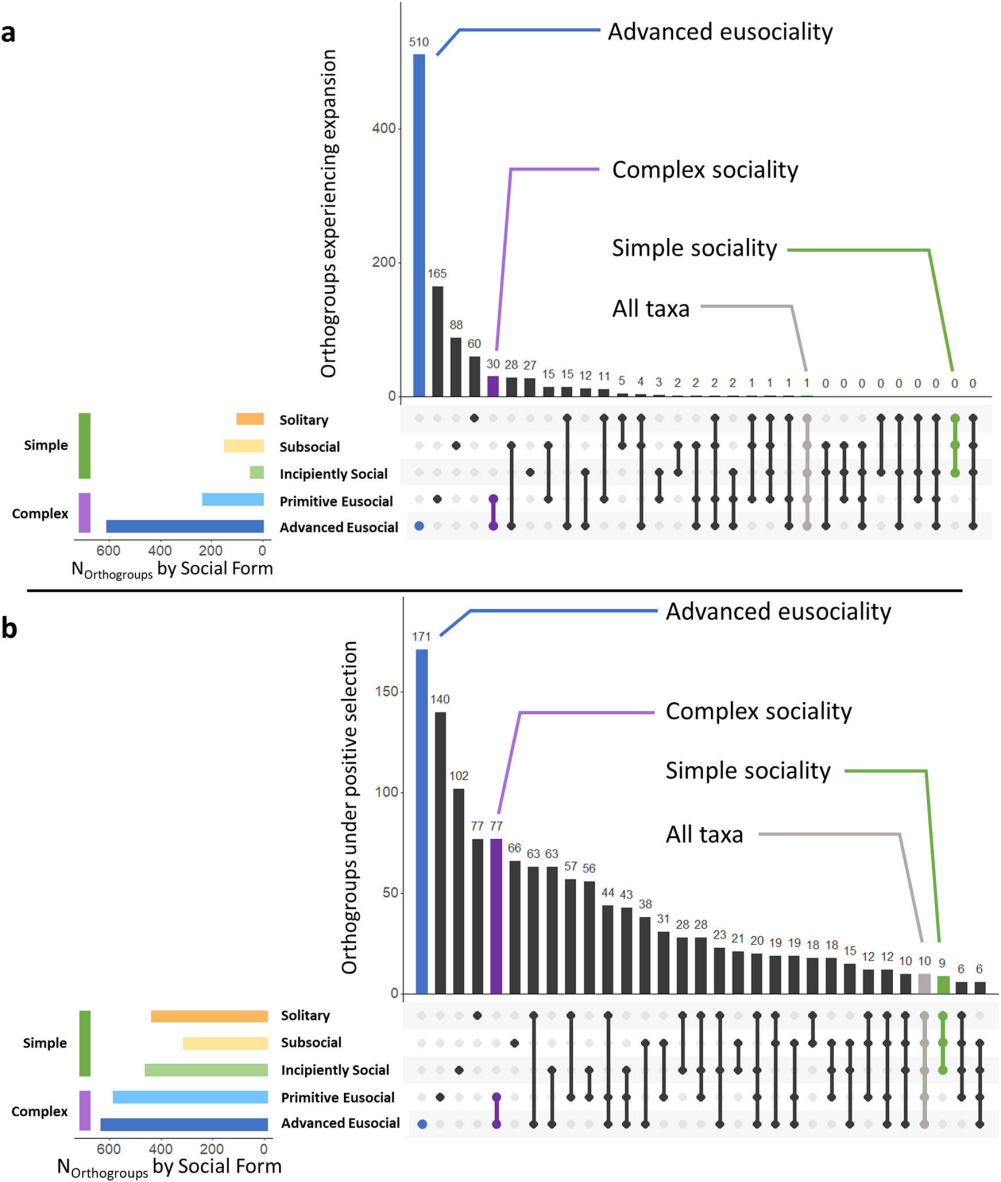
UpSet charts displaying counts and uniqueness of orthologous gene families experiencing expansion or positive selection by social complexity. Dots and lines in bottom right indicate unique or shared membership of orthogroups among social groups; vertical columns indicate total orthogroup counts for those categories. Colored lateral columns indicate total counts of orthogroups by sociality from solitary (orange) through advanced eusocial (dark blue); simple (green), complex (purple), and all social forms (gray) are also specified. **a** Counts and uniqueness of orthologous gene families experiencing significant expansion (*p* < 0.05) both increase dramatically with derivations in social complexity. **b** Similarly, counts and uniqueness of orthogroups under significant positive selection (d*N*/d*S* > 0; *p* < 0.05) increase with derivations in social complexity.

A total of 10,355 orthologous gene families were identified among our 16 lineages (Fig. 1a) during CAFE analysis^45^, of which 2,036 were found to be significantly expanded in at least one lineage (Data S4, S5). Overall, counts of significantly expanded gene families increased dramatically with social complexity, from 60 groups uniquely expanded across our solitary species to 510 uniquely expanded among our advanced eusocial taxa (Data S4, S7; Fig. 2a). Evolutionary derivations in social complexity are expected to be accompanied by functional elaborations and expansions among gene families as pleiotropic constraints are removed during the emergence of castes^2,15^. Our results provide support for this prediction and corroborate previous comparative genomic analyses^4^. Among notable gene families that followed this trend were the 7 transmembrane (tm) and 7tm Odorant receptor domain-containing genes. Chemosensory capability is critical for navigation and resource acquisition among insects^46^, and plays an important role in the continuous, caste-based communication of socially complex Hymenoptera^47,48^.

An additional 247 families were uniquely expanded among our six xylocopine species (*N*_total_ = 2283). Within the Xylocopinae, a total of 19 gene families were significantly expanded across all Xylocopinae except the solitary *Ct. terminalis*, including gene families for reverse transcriptases, homeobox transcription factors, and two Immunoglobulin I-sets (Data S6). Two families, the cytochrome P450 and cadherin domains, were expanded specifically in our eusocial xylocopine species. Cytochrome P450 and cadherin domains likely play important roles in detoxification, chemical communication, and immune function^49^; and affiliated genes have been repeatedly and consistently implicated in the operation of derived forms of social nesting across Hymenoptera^50^. Capacities for chemical communication and immune resistance are critical among advanced eusocial Apinae^51^. As has been noted across ants and other eusocial lineages^5^, these data suggest that affiliated expansions in the P450 and related gene families may also be important during the evolution of social complexity in the Xylocopinae.

### Positive selection

#### Rates of protein evolution are tied to social complexity rather than phylogenetic lineage

**Fig. 3:**
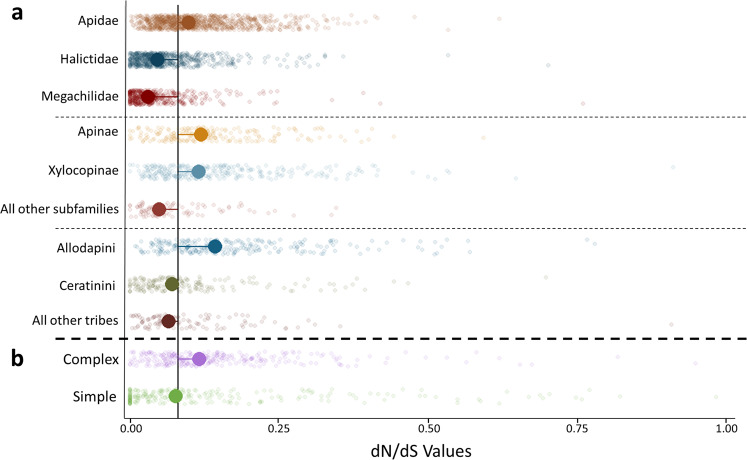
Dot plots displaying rates of protein change (d*N*/d*S*) among orthogroups calculated across major phylogenetic and social phenotypic tiers. Dots are jittered for visualization; vertical black line indicates global median d*N*/d*S* value; larger colored dots indicate group-specific median values (full lists of d*N*/d*S* values can be found in Datas S23-S38). **a** At every phylogenetic level inspected, lineages containing highly social lines (e.g. Apidae; Apinae and Xylocopinae; Allodapini) feature significantly higher rates of protein evolution (Data S45). **b** Orthogroups associated with more complex forms of sociality (i.e. Primitive and Advanced Eusociality) experience significantly higher rates of protein evolution than less derived forms.

Phylogenetic Analysis by Maximum Likelihood (PAML v 4.9^52^) was used to determine whether gene orthogroups may be undergoing positive selection (i.e. elevated non-synonymous over synonymous mutations; d*N*/d*S* > 1, *p* < 0.05) at either the lineage or social phenotypic levels and thus likely operating with some evolutionary consequence. We identified a total of 1460 orthogroups experiencing significant positive selection by lineage (Data S20–S31) and 1302 by sociality (Fig. 2b; Data S32–S36). Here, we discuss PAML results with regard only to branches under positive selection. At the family level, Apidae contained the greatest number of taxonomically restricted orthogroups under positive selection (*N* = 313, Fig. 3a, S7; Data S23–S25), and the highest average rates of protein evolution (d*N*/d*S* values) of any family considered (Data S45). Notably, functional enrichment among these orthogroups including both aromatic and organic cyclic compound metabolism, suggests additional support for the role of chemical communication within this family (Data S40; 48). Within Apidae, the two subfamilies containing advanced eusocial taxa also featured both the largest numbers of orthogroups under positive selection (Xylocopinae, *N* = 293, Data S27; and Apinae, *N* = 196, Data S26) and comparable rates of protein evolution (Wilcoxon test, *Z* = 0.26, *p* = 0.7892; Fig. 3a, S7), both of which were significantly higher than non-eusocial subfamilies (Data S45). Evidence of increased protein evolution was also observed within Xylocopinae. Tribe Allodapini featured both the greatest counts of orthogroups under positive selection (*N* = 246, Figs. 3a, S7; Data S30) and significantly elevated overall rates of protein evolution compared to both sister tribe Ceratinini (Wilcoxon test, *Z* = 8.01, *p* = 1.10E−15) and all remaining species (Data S45). Regardless of lineage, increases in social complexity accounted for greater numbers of novel orthogroups under positive selection (unique OGs, *N*_solitary_ = 77 through *N*_advanced eusocial_ = 171; χ^2^ = 74.33, d*f* = 4, *p* = 0; Fig. 2b; Data S39) and higher rates of protein evolution (d*N*/d*S* values; Wilcoxon test, *Z* = −4.314, *p* < 0.0001; Fig. 3b; Data S45). Interestingly, orthogroups under positive selection in eusocial lineages were uniquely functionally enriched for oxidoreductase activity (Data S40). Mitigation of oxidative damage is likely a critical component of caste longevity across eusocial insects, which typically feature exceptionally long-lived queens^53^. Taken together, our results provide clear evidence of both quantitatively and qualitatively greater measures of positive selection on larger sets of taxonomically restricted genes across two independent origins of eusociality^2^. They also present additional empirical support for the theory that positive selection will operate with increasing intensity as lineages become more socially complex^4,15^. Notably, our results also reveal that positive selection appears to operate with considerable consistency both within and across independent social lineages.

### Comparative transcriptomics

#### Elevated protein evolution specifically among genes associated with caste-like roles

Differentially expressed genes (DEGs) are expected to play a major role in the emergence and elaboration of social traits^3^ and thus to become the targets of positive selection as social traits establish^15^. Within Xylocopinae, we found that the majority of DEGs under significant positive selection showed overexpression in non-reproductive individuals (*N*_reproductive_ = 10 vs *N*_non-reproductive_ = 22, 69%) and foragers (*N*_foraging_ = 20 vs *N*_waiting_ = 12; Data S8–S11; Fig. S8). We also found that significantly more non-reproductive DEGs were under positive selection than expected by chance in both of the eusocial allodapine species (Data S39). Across all 16 bee genomes, the number of genes under positive selection also increased with species social complexity (Fig. 2b). These results provide further empirical support from outside Apinae for the role of elevated protein change specifically among DEGs associated with non-reproductive and/or foraging roles^4,36,38^.

#### Differentially expressed genes associated with carpenter bee sociality are ancient

Phylostrata analysis (via phylostratR v 0.20^54^) was used to assign a total of 20,405 orthologous gene groups to twenty levels of taxonomic constraint based on orthogroup evolutionary age (Data S18). Across lineages, most orthogroups were assigned to older levels (i.e., cellular organisms through Insecta, 65%). Although the overall majority of differentially expressed genes were also ancient, they were significantly overrepresented at older levels in our ceratinine species (cellular to Insecta vs Hymenoptera to tribe; χ^2^_Ceratinini_ = 20.63, d*f* = 1, *p* = 5.57e−6; Fig. S6; Data S8–11; S19). It thus appears that while taxonomically restricted genes are thought to play an important role in the expression of derived sociality among some lineages (*e.g*., *A. mellifera*^55^; Formicidae^5^) our data indicate that this may not be the case among social Xylocopinae.

#### Gene expression, enrichment, and regulatory consistencies by social phenotype rather than shared lineage

The toolkit hypothesis suggests that conserved differentially expressed genes likely play consistent underlying roles in the emergence and expression of similar social traits across taxa^18^. Accordingly, the 396 differentially expressed genes identified across our xylocopine taxa (Data S8–S12) included notable homologs (determined by BLASTn with shared gene identity ≥ 70% and *p* < 1.0E-5) expressed in phenotypically consistent contexts across other lineages (Fig. S9; Data S14–17). For example, DEGs associated with queens or workers of advanced eusocial *E. tridentata* (e.g. *Troponin C*) were also differentially regulated in comparable roles (i.e. foragers) among other advanced eusocial bees (e.g. *A. mellifera*, Fig. S9) and ants (e.g. *T. longispinosus*, *S. invicta*; Data S17). These results signal additional support for the role of differentially expressed and deeply conserved genes in the regulation of insect social traits^18,28,56^.

**Fig. 4:**
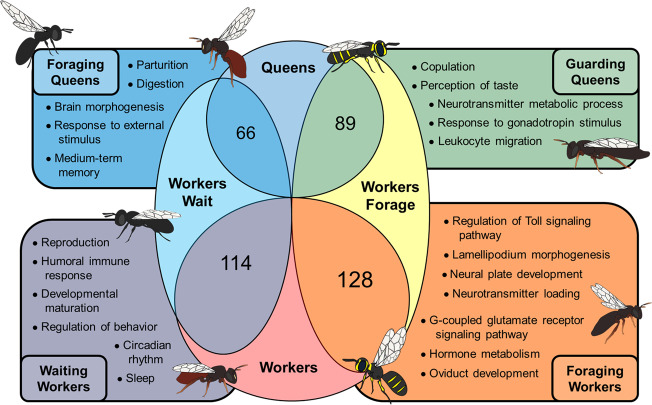
Comparison of neural, metabolic, and immune-associated GO terms significantly enriched (*p* < 0.05; dispensability < 0.50) among queens and workers by shared phenotype. Circle overlap sizes are equivalent to relative proportions of total GO terms uniquely associated with each phenotype considered; images are illustrative of foraging and guarding/waiting behavior by queens and workers. Enrichment for reproduction was detected in all groups except for foraging workers, which instead featured more enrichment for immune activity. The full list of GO terms can be found in Data S41.

Despite occupying separate phylogenetic lineages within Xylocopinae, there were consistencies in gene ontological (GO) enrichment among our ceratinine and allodapine taxa by whether workers waited on or foraged for the nest (Fig. 4; Data S41). For example, non-reproductive females of *C. australensis* and *E. robusta* were significantly enriched for reproductive activity (e.g. reproduction), directly corroborating the “workers wait” strategy of attempting to lay eggs and eventually superseding as the reproductive dominant of the nest^57,58^. By contrast, the foraging-focused workers of *C. japonica* and *E. tridentata* were instead enriched for immune (e.g., regulation of Toll signaling pathway) and neural functions, processes that are likely important for individuals that spend most of their time foraging for the nest along the lines of other derived worker castes^26,27,59^.

**Fig. 5:**
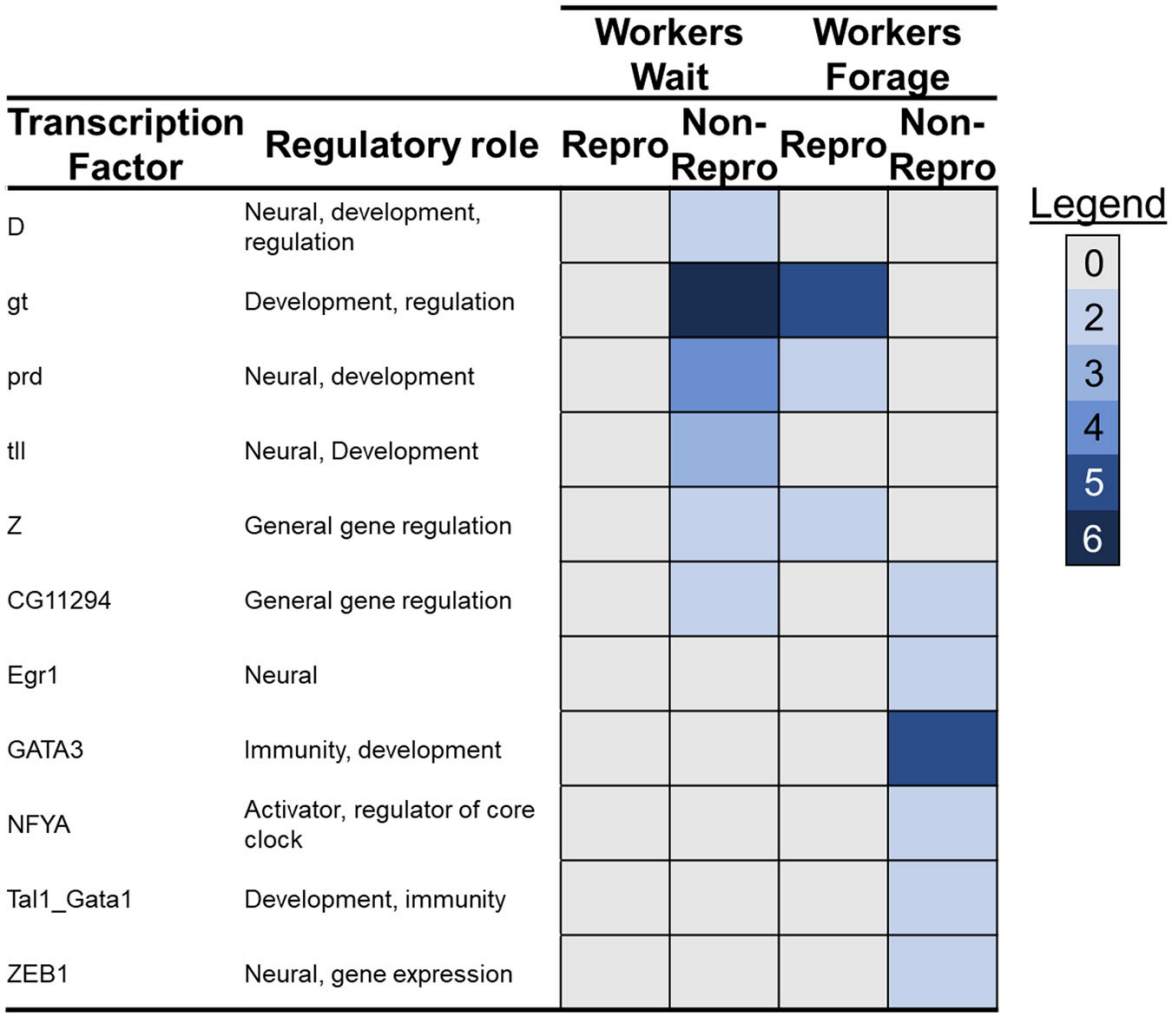
Heat map highlighting TFBS motifs with neural, immune, or developmental roles, significantly enriched upstream of genes upregulated in workers that wait or forage regardless of lineage (full list in Data S42). Motif names and broad regulatory involvement are provided. Enrichment counts for each motif in the upregulation of genes associated with each phenotype is then indicated by color intensity (see legend: gray—no enrichment, blue—enriched in both species, with darker blues indicating greater enrichment).

In comparing predicted regulatory elements related to each taxon’s DEGs, we found a trend of increased overall counts with increasing social complexity; i.e. 88 transcription factors (TFs) in incipiently social *C. australensis* (44 of which were unique to *C. australensis*) to 396 TFs in advanced eusocial *E. tridentata* (of which 304 were unique; Data S42). *Exoneurella tridentata* and *E. robusta*, both eusocial, were also enriched for significantly more TFs than expected given DEG counts (*N*_DEGs_ vs *N*_TFBS_ by Species; χ^2^-test, χ^2^ = 117.70, d.f. = 3, *p* < 0.00001). Comparing TFs enriched in common among taxa, significantly more were shared among non-reproductive females that demonstrated similar social phenotypes than among those that shared a lineage (*N*_Phenotype_ = 18 vs *N*_Lineage_ = 4 vs *N*_NotShared_; χ^2^ = 8.09, d*f* = 1, *p* = 0.004; Fig. 5; Data S42, S43). However, this was not found among reproductive females (*N*_Phenotype_ = 15 vs *N*_Lineage_ = 13 vs *N*_NotShared_; χ^2^ = 0.123, d*f* = 1, *p* = 0.73). TFs enriched among non-reproductives that wait on the nest were associated primarily with development (e.g. *D*, *tll*) and included those which were also enriched among the reproductive individuals of *C. japonica* and *E. tridentata* (e.g. *gt*, *prd*, and *z*). *Gt*, *prd*, and *z* are functionally associated with neural development (including chemosensation) and epigenetic regulation of gene expression and have all been previously associated with guarding behavior in *C. calcarata*^34^. Non-foraging individuals often act as nest guards, either while waiting to supersede the nest, or to ensure the survival of their own brood^28,57,58^. As such, *gt*, *prd*, and *z* may play conserved regulatory roles in the induction of guarding behavior among social lineages^3^. By contrast, workers that forage shared more functionally diverse regulatory enrichment, including TFs involved in development (e.g. *NKX3-1*; *TFAP2a*), learning, circadian rhythm, and memory (e.g. *Egr1*, *NFYA*, *ZEB1*), and immunity (e.g. *GATA3; Tal1_Gata1*; Fig. 5). Further, six TFs from this set were previously associated with pre-reproductive foraging in *C. calcarata*^34^ including *Egr1*, *GATA3*, *NFYA*, and *Tal1_Gata1*, associated with learning, memory, and immune function. Of particular note from this set is *early growth response protein 1* (*Egr1*), previously found to have a widely-conserved role in socially responsive gene regulation^60^ including a critical role in honey bee foraging^61^, and recently proposed as a candidate TF for tasks involving time-memory^62^. Taken together, these results support the suggestion that regulatory networks underlying social behavioral phenotypes may be broadly convergent across lineages^5,36^. Our data also corroborate previous observations that regulatory network scale and complexity tend to increase as lineages evolve greater degrees of sociality^4,5,63^, reinforcing the importance of regulatory expansion and elaboration during the evolution of sociality.

## Conclusions

In this study, we present newly sequenced genomes and transcriptomes of four carpenter bees (Apidae: Xylocopinae) and combine these data with published resources from 12 additional bee species to perform the most comprehensive comparative assessment of social evolution in bees to date. Our data provide a chance to carefully compare two independently evolved and ecologically distinct social bee lineages; and an unprecedented opportunity to examine mechanisms of social evolution within an understudied and socially diverse eusocial lineage (Xylocopinae). Ultimately, our study finds clear empirical support for the predictions of the social ladder framework: gene family expansions, protein evolution, and regulatory element assortment are consistently extended among increasingly complex social lineages^2,19^. Differentially expressed genes are deeply conserved and evolutionarily ancient; and gene regulatory and functional elements appear to play highly conserved roles in the expression of particular social phenotypes (e.g. foraging behavior) across lineages^5,18,64^. More broadly, despite independent origins of eusociality, members of at least two different bee lineages appear to have similar evolutionary signatures of social complexity as a result of gene family expansions and increasingly strong positive selection on key proteins, differentially expressed genes, and regulatory elements. It therefore appears that sociality itself, more than phylogenetic inertia, shapes the evolutionary trajectory of social lineages. At present, available data offer abundant evidence in support of the applicability of the social ladder framework and highlight the importance of social evolution as a major and surprisingly consistent sculptor of genomic change among bees^1,2,6^. Future studies across additional independent origins of sociality that consider the great diversity of social taxa are necessary to further test the ubiquity and importance of what appear to be key molecular mechanisms of evolutionary change towards group living.

## Methods

### Sample collection and preparation

Four bee species were collected for new genomes and transcriptome analyses. The primitively eusocial *Exoneura robusta*^57^ and advanced eusocial *Exoneurella tridentata*^59^ were collected from the Dandenong Ranges and Lake Giles, Australia respectively. The primitively eusocial *Ceratina japonica*^26,27^ was collected from Sapporo, Japan, and solitary *Ctenoplectra terminalis* was collected in Kakamega, Kenya. Details on sampling and preservation protocols can be found in supplementary materials.

### Genome sequencing and analysis

Whole body genomic DNA was extracted using phenol-chloroform extraction and submitted to Genome Quebec for cleanup, library preparation, and Illumina shotgun sequencing. To improve genome assembly, DNA samples were also used to construct 150 bp mate pair and 100 bp single strand libraries and sequenced on an Illumina HiSeq 2500. Before filtering, genome sequencing produced a total of 139 Gb of raw sequence data across our six species (with an average of 34.8 GB, 39.7 million reads at 33x coverage per species). Prior to assembly, filtering removed low quality reads, reads with a high proportion of Ns or poly-A sections, and reads for which mate pair ends overlapped or were merged. Each genome was then assembled and annotated before being assessed for completeness in relation to the *A. mellifera* genome. All newly generated genomic data can be found using NCBI BioProject numbers PRJNA413373, 526224, 413974, and 526241 (Data S1). De novo genome data were then combined with published genomic data from twelve additional bee species (Data S2, Fig. 1, S4) and aligned for comprehensive comparative analyses of gene family expansions (CAFE, Figs. S1–3; 45), evidence of molecular evolution (PAML^52^), and gene ages (phylostratR^54^). Additional test details are provided in supplementary methods.

### Transcriptome sequencing and analysis

RNA was extracted from whole heads of queens and workers of *C. japonica*, *E. tridentata*, and *E. robusta*, and solitary females of *Ct. terminalis* and submitted for library prep and paired-end Illumina HiSeq 2500 sequencing (Genome Quebec). Read data were aligned to species genomes before being used for analysis (accessible under PRJNA413373, 526224, 413974, and 526241; Data S1). Significantly differentially expressed genes (DEGs; adjusted *p*-value < 0.05) were identified using DESeq^65^ and corroborated by DESeq2^66^. Results of DEG analysis were then used to inform analyses of gene ontology (GO) term (topGO v3.7^67^) and transcription factor binding site (TFBS) motif enrichment (cis-Metalysis pipeline^68,69^), and comparative analyses of biological contexts of differential gene expression between newly sequenced xylocopine species and 24 additional studies (Data S32–36).

Additional details on all methods employed for transcriptome analysis can be found in supplementary materials.

## Supplementary information

Supplementary Information

Description of Additional Supplementary Files

Supplementary Data 1

## Data Availability

All newly generated genomic and transcriptomic data used in this study can be freely accessed via NCBI BioProject numbers PRJNA413373, 412093, 526224, 413974, and 526241.
