# Supplementary material for: Sociality sculpts similar patterns of molecular evolution in two independently evolved lineages of eusocial bees
Source: Commun Biol. 2021 Feb 26;4:253. doi: 10.1038/s42003-021-01770-6 (PMC7977082; doi:10.1038/s42003-021-01770-6)
Supplement: Supplementary file 1 — Supplementary Information [file 42003_2021_1770_MOESM1_ESM.pdf]

SUPPLEMENTAL METHODS AND MATERIALS

Genome sequencing and assembly continued

For each species, reads were checked for quality using FastQC (1) before using Trimmomatic to remove adapter contamination of Illumina Nextera-related adapter sequences (2). FastUniq was then used to excise duplicates from each library (3) and errors in the reads were then corrected using the SOAPdenovo error correction module (4) using a kmer size of 31. This process left an average of 394 Mb of filtered sequence data (**Data S2**, **S3**).

Putatively misassembled scaffolds were then broken down at weak points using REAPR (5). These broken scaffolds/contigs were then reassembled using SSpace (6) and assembly gaps left after scaffolding were filled using Gapfiller (7). Next, L_RNA_Scaffolder (8) and Trinity (9) were used to further scaffold contigs/scaffolds that were broken in gene annotations. This was followed by a final round of gap filling using GapFiller (7).

Genome annotation and functional enrichment continued

For each genome, gene annotations were performed using the MAKER2 pipeline (10); along with the gene prediction algorithms SNAP (11), Augustus (12), and GeneMark (13). Annotations with a MAKER AED score over 0.75 or with no significant InterProScan domains were flagged as weakly supported (10). RepeatMasker was then used, with default parameters, to identify repetitive elements based on the Repbase transposable element (TE) library (‘species=all’) along with a custom species-specific repeat library for each genome, generated with RepeatModeler (14). Identified genes were then functionally annotated using BLAST2GO (15) with default settings based on queries of the NR database with BLASTP (e-value cutoff of 1

e-5) and all significant InterProScan hits. The KAAS server was then used to assign KEGG orthology (16).

The completeness of each genome's assembly and annotation was then assessed using the OrthoDB Benchmarking Universal Single-Copy Orthologs (BUSCOs) pipeline (17), which tested for the presence and completeness of orthologs in relation to *A. mellifera* sequences from arthropod-level BUSCOs in each species (i.e. *C. japonica*, *E. robusta*, *E. tridentata*, and *Ct. terminalis*; **Data S3**, **Figure 1B**).

Transcriptome sequencing and assembly continued

RNA was extracted from whole heads of three queens and three workers of *C. japonica* and *E. tridentata*, five queens and workers of *E. robusta*, and three female *Ct. terminalis* (**Data S1**). Heads were removed on dry ice and immediately processed using the QIAGEN RNeasy Kit and protocol (Cat # 73404). After RNA quality was confirmed on an Agilent Tape Station 2200, samples were submitted for library prep and paired-end Illumina HiSeq 2500 sequencing (Genome Quebec). Each species' read data were then aligned to their respective genomes before being used for analysis. Raw read data can be accessed under project numbers PRJNA413373, 526224, 413974, and 526241 (**Data S1**).

Gene family analyses (CAFE)

De novo genome data was then combined with published genomic data from twelve additional species, including *Ceratina calcarata* (18), *C. australensis* (19), *Apis florea* (20), *A. mellifera* (21), *Bombus impatiens*, *B. terrestris* (22), *Lasioglossum albipes* (23), *Megachile rotundata*, *Dufourea novaeangliae*, *Habropoda laboriosa*, *Eufriesea mexicana*, and *Melipona*

quadrifasciata (24; **Data S2**). Genome data were aligned and used to infer a maximum-likelihood species tree in RAxML v8.9 (25) [*tree ((Dnov:85,Lalb:85):30,(Mrot:107,((Cteno:91,((Etrid:36, Erob:36):21,(Caust:55,(Cjap:45,Ccalc:45):10):2):34):9,(Hlab:87,(Emex:81,((Mqua:68,(Bimp:13,Bter:13):55):10,(Amel:22,Aflo:22):56):3):6):13):7):8)*]. Orthologous gene groups were then identified in all 16 bee species by first isolating the longest transcript isoform (with a minimum 50 amino acid sequences) for each gene. Filtered gene sets were then assigned to orthologous groups (i.e. gene families) using OrthoMCL (26) via an all-against-all BLASTP comparison using default settings (p-value cut-off of 1.0 e-05). CAFE v3 (27) was then used to estimate birth and death rate (lambda) for each identified gene family; and significant contractions/expansions were identified using a family-wide p-value cut-off of 0.05 (**Figures S1-S3; Data S4**). The command lambda -s was used to search for the optimized lambda across each branch, with parameters allowing lambda to vary by social organization. Significant gene family expansions were then analyzed for gene ontology enrichment using a Fisher's exact test in the R package topGO (28; **Data S5-7**).

Molecular evolution analyses – (PAML)

Orthology assignment: In addition to the four newly sequence genomes, 12 previously published reference genomes and annotation data were downloaded from BeeBase (29-31) and NCBI (32) (**Figure S4**). The predicted protein sequences for each species were downloaded or extracted from the annotation files (GFF) for ortholog prediction. OrthoMCL (v2.0.9) (26), was used for orthology assignment, using an inflation value of 2.5. To prepare for downstream

comparative analyses, identified orthogroups were then filtered to retain only groups which i) featured a single copy gene for each species and ii) were present in at least 10 of the 16 genomes.

Phylogenetic analyses: To align each orthogroup peptide sequence, we ran GUIDANCE (v. 2.02) (33), with the alignment algorithm set to MAFFT (34). Peptide sequences were then reverse translated to obtain codon alignments as implemented by PAL2NAL (v14.0) script (35). We set seqType to "nuc" and proc_num to 4, but left all other parameters on default settings. The input order of the sequence was maintained in the final alignment. The aligned sequences were then used for phylogenetic analyses. RAxML-HPC (v8.9) (25) was then used to perform maximum likelihood (ML) analyses with algorithm selection set to compute rapid Bootstrap analysis for selecting the best-scoring ML tree in one program run. We set the number of alternative runs on distinct starting trees to 100 and selected GTR + gamma as an evolutionary model for DNA partition analysis (36).

dN/dS calculations: We used the codeml program of the PAML (v 4.9) (37) package to calculate dN/dS (tests for neutral selection and tests for positive selection) among the various groups within the phylogenetic tree (**Data S22-S38**). The better fitting model was determined by comparing the Log Likelihood Ratios (LRT) using a *p*-value threshold of 0.05. The LRT comparison for models M0 (NSsites = 0, model = 0) and M1 (NSsites = 1, model = 0), determined if the sites have reasonable average omega or not. For the genes that do not have one ratio, LRT comparison for M1 and M2 (NSsites = 2, model = 0) was performed to determine positive selection within branches. These genes were also assessed via models M7 (NSsites = 7, model = 0) and M8 (NSsites = 8, model = 0) to identify sites under positive selection.

Phylostrata

Phylostratiography analyses were performed on all orthogroups identified during preparation for PAML using phylostratR (v0.20) (38). The NCBI taxa IDs of the 16 included species were used in the program to automatically fetch proteomes from the UniProt database (39). For each ancestral node, a subset of related species (n=5) and a program-selected suite of bacterial proteomes from UniProt were used for analysis. BLASTp (40) was then performed against all the other proteomes to build the database; homology of each focal gene was then derived (1e-5 e value cut off) and stratification results were compiled. There is some debate about the biological accuracy of the phylostratigraphic analysis (see 41, 42 and references therein); specifically, as phylostratR relies on BLAST, any variation in sequence data – from true mutations or sequencing error – may be interpreted as biologically meaningful. As such, phylostratR may slightly underestimate gene ages, though this technical error rate has been defended as not substantial enough to invalidate the informative value of the analysis.

Differential gene expression

Brain gene expression data from four focal species, *C. australensis*, *C. japonica*, *E. robusta*, and *E. tridentata,* were used to perform several analyses of gene expression and regulation. These species represent a pairwise comparison of xylocopine species in which workers that wait (i.e. *Ceratina australensis*; *Exoneura robusta*) versus those that forage for their nestmates (i.e. *Ceratina japonica*; *Exoneurella tridentata*). DESeq (43) was used in R v3.2.4 to identify significantly differentially expressed genes (DEGs; p-value adjusted < 0.05; corroborated by DESeq2; 44) associated with reproductive status (reproductive vs non-reproductive) or foraging behavior (foraging vs non-foraging) in each species. Gene ontology (GO) term enrichment was then determined for all upregulated DEG lists using topGO v3.7 (45);

and significantly enriched GO term lists ($p < 0.05$) were further condensed for exploration using Revigo to focus on parent terms with a dispensability rating ≤ 0.5 (46). GO terms were then explored and compared among species based on shared phylogeny (Ceratinini vs Allodapini) and shared phenotype (workers wait vs workers forage). GO term enrichment was also determined for all single-copy *A. mellifera* genes utilized during OG assignment for PAML using biomartr (47) to secure GO data from ensembl.

Transcription factor binding motifs

We used the cis-Metalysis pipeline (48, 49) to run an analysis of transcription factor binding site (TFBS) motif enrichment among all DEGs determined via DESeq analysis for each species, accessing the JASPAR Insect and Vertebrate databases for motif reference (50). To prepare inputs for this analysis, the relative regulatory direction of each DEG was calculated using Z-scores across each biological condition. A Z-score provides an accurate measure of positive or negative standard deviations away from average expression: meaning a score near 0 indicates little difference from average expression, and positive and negative values respectively indicate up- and down-regulation relative to the average. Using this logic, DEGs with Z-scores less than or equal to -0.333 were considered as down-regulated; greater than or equal to -0.333 but less than or equal to 0.333 were considered non-differentially regulated from the average; and those with a Z-score greater than 0.333 were considered up-regulated in that condition. These calculated regulatory directions were then confirmed for consistency against results of DESeq analyses. These regulatory directional values were taken together with promoter region sequence data drawn from 5kb windows upstream of all genes for which sequence and expression data were available and fed into Stubb to identify TFBS motif enrichment. Stubb

scores both individual and compound TFBS motifs for each biological context based on motif presence in each context-associated gene's promoter region and the defined regulatory direction of each gene. Total Stubb outputs were then fed through cis-Metalysis to select the top 1% most significantly enriched portion of the results to produce a list of best-supported TFBS motifs associated with up- and down-regulated genes in each condition. Given the exhaustive permutation testing of the cis-Metalysis pipeline we consolidated extensive resulting TFBS motif lists for further analysis by focusing only of functionally non-redundant outputs (e.g. while the outputs "not A and B" and "just B" are technically distinct, they are effectively both functionally reducible to "just B;" whereas the motif outputs "A and B" and "A and C" are both distinct pairs, and would thus both be kept for further analyses).

Comparative Analyses

We performed independent BLASTn searches (1e-5 e value cut-off) between the *C. japonica*, *C. australensis*, *E. robusta*, and *E. tridentata* genomes against 21 additional genomic and transcriptomic datasets (representative of seven bees: *Apis mellifera, Bombus impatiens, Bombus terrestris, Euglossa dilemma, Ceratina calcarata, Ceratina australensis, Megalopta genalis*; three ants: *Solenopsis invicta, Temnothorax longispinosus, Harpegnathos saltator*, two wasps: *Polistes metricus, Polistes canadensis*, and the fly *Drosophila melanogaster*; **Data S32**). Resulting lists of homologous genes were then used to compare gene differential expression contexts between each ingroup species and 24 additional studies collectively exploring variations in reproductive status, caste, and behavior in Hymenoptera (**Data S32 – S36**). Wherever suitable data were available, similar methods were used to compare enriched GO terms and TFBS motifs (**Data S11**, **S12**).

References

1) Patel, R.K., Jain, M. NGS QC Toolkit: a toolkit for quality control of next generation sequencing data. *PloS One* **7,** e30619 (2012).

2) Bolger, A.M., Lohse, M., Usadel, B. Trimmomatic: a flexible trimmer for Illumina sequence data. *Bioinformatics* **30,** 2114-2120 (2014).

3) Xu, H. *et al*. FastUniq: a fast de novo duplicates removal tool for paired short reads. *PloS One* **7,** e52249 (2012).

4) Luo, R. *et al*. SOAPdenovo2: an empirically improved memory-efficient short-read de novo assembler. *Gigascience* **1,** 2047-217X (2012).

5) Hunt, M., *et al*. REAPR: a universal tool for genome assembly evaluation. *Genome Biology* **14,** R47 (2013).

6) Boetzer, M., Pirvano, W. Toward almost closed genomes with GapFiller. *Genome Biology* **13,** R56 (2012).

7) Boetzer, M., Henkel, C.V., Jansen, H.J., Butler, D., Pirovano, W. Scaffolding pre-assembled contigs using SSPACE. *Bioinformatics* **27,** 578-579 (2011).

8) Xue, W., *et al*. L_RNA_scaffolder: scaffolding genomes with transcripts. *BMC genomics* **14,** 604 (2013).

9) Haas, B.J. *et al*. De novo transcript sequence reconstruction from RNA-seq using the Trinity platform for reference generation and analysis. *Nature Protocols* **8,** 1494 (2013).

10) Holt, C., Yandell, M. MAKER2: an annotation pipeline and genome-database management tool for second-generation genome projects. *BMC bioinformatics* **12,** 491 (2011).

11) Korf, I. Gene finding in novel genomes. *BMC bioinformatics* **5,** 59 (2004).

12) Stanke, M., Waack, S. Gene prediction with a hidden Markov model and a new intron submodel. *Bioinformatics* **19,** ii215-ii225 (2003).

13) Lukashin, A.V., Borodovsky, M. GeneMark. Hmm: new solutions for gene finding. *Nucleic Acids Res.* **26,** 1107-1115 (1998).

14) Jurka, J. *et al*. Repbase update, a database of eukaryotic repetitive elements. *Cytogenetic and genome research* **110,** 462-467 (2005).

15) Conesa, A. *et al*. Blast2GO: a universal tool for annotation, visualization and analysis in functional genomics research. *Bioinformatics* **21,** 3674-3676 (2005).

16) Moriya, Y., Itoh, M., Okuda, S., Yoshizawa, A.C., Kanehisa, M. The metabolic pathways were examined through KAAS (KEGG automatic annotation server): an automatic genome annotation and pathway reconstruction server. *Nucleic Acids Res*. **35,** W182-W185 (2007).

17) Simão, F.A., Waterhouse, R.M., Ioannidis, P., Kriventseva, E.V., Zdobnov, E.M. BUSCO: assessing genome assembly and annotation completeness with single-copy orthologs. *Bioinformatics* **31,** 3210-3212 (2015).

18) Rehan, S.M., Glastad, K.M., Lawson, S.P., Hunt, B.G. The genome and methylome of a subsocial small carpenter bee, Ceratina calcarata. *Genome Biol. Evol.* **8,** 1401-1410 (2016).

19) Rehan, S.M. *et al*. Conserved genes underlie phenotypic plasticity in an incipiently social bee. *Genome Biol. Evol.* **10,** 2749-2758 (2018).

20) Wang, Y. *et al*. Regulation of behaviorally associated gene networks in worker honey bee ovaries. *J. Exp. Biol.* **215,** 124-134 (2012).

21) Weinstock, G.M. *et al*. Insights into social insects from the genome of the honeybee Apis mellifera. *Nature* **443,** 931-949 (2006).

22) Sadd, B.M. *et al*. The genomes of two key bumblebee species with primitive eusocial organization. *Genome Biology* **16,** 76 (2015)

23) Kocher, SD. *et al*. The draft genome of a socially polymorphic halictid bee, Lasioglossum albipes. *Genome Biology* **14,** R142 (2013).

24) Kapheim, K.M. *et al*. Genomic signatures of evolutionary transitions from solitary to group living. *Science* **348,** 1139-1143 (2015).

25) Stamatakis, A. RAxML-VI-HPC: maximum likelihood-based phylogenetic analyses with thousands of taxa and mixed models. *Bioinformatics* **22,** 2688-2690 (2006).

26) Li, L., Stoeckert Jr., C.J., Roos, D.S. OrthoMCL: identification of ortholog groups for eukaryotic genomes. *Genome Res* **13,** 2178-2189 (2003)

27) De Bie, T., Cristianini, N., Demuth, J.P., Hahn, M.W. CAFE: a computational tool for the study of gene family evolution. *Bioinformatics* **22,** 1269-1271 (2006).

28) Alexa, A., Rahnenführer, J., Lengauer, T. Improved scoring of functional groups from gene expression data by decorrelating GO graph structure. *Bioinformatics* **22,** 1600-1607 (2006).

29) Elsik, C.G. *et al*. Hymenoptera Genome Database: integrating genome annotations in HymenopteraMine. *Nucleic Acids Res* **44,** D793-800 (2016).

30) Elsik, C.G., Tayal, A., Unni, D.R., Burns, G.W., Hagen, D.E. Hymenoptera genome database: using HymenopteraMine to enhance genomic studies of hymenopteran insects. Pp513-556 in *Eukaryotic Genomic Databases* (Humana Press, New York, NY, 2018).

31) Munoz-Torres, M.C. *et al.* Hymenoptera Genome Database: integrated community resources for insect species of the order Hymenoptera. *Nucleic Acids Res* **39,** D658-662 (2011).

32) O'Leary, N.A. *et al.* Reference sequence (RefSeq) database at NCBI: current status, taxonomic expansion, and functional annotation. *Nucleic Acids Res* **44,** D733-745 (2016).

33) Sela, I., Ashkenazy, H., Katoh, K., Pupko, T. GUIDANCE2: accurate detection of unreliable alignment regions accounting for the uncertainty of multiple parameters. *Nucleic Acids Res* **43,** W7-14 (2015).

34) Katoh, K., Standley, D.M. MAFFT: iterative refinement and additional methods. *Methods Mol Biol* **1079,** 131-146 (2014).

35) Suyama, M., Torrents, D., Bork, P. PAL2NAL: robust conversion of protein sequence alignments into the corresponding codon alignments. *Nucleic Acids Res* **34,** W609-612 (2006).

36) Chai, J., Housworth, E.A. On Rogers' proof of identifiability for the GTR + Gamma + I model. *Syst Biol* **60,** 713-718 (2011).

37) Yang, Z. PAML 4: phylogenetic analysis by maximum likelihood. *Mol Biol Evol* **24,** 1586-1591 (2007).

38) Arendsee, Z. *et al.* phylostratr: A framework for phylostratigraphy. *Bioinformatics* **35,** 3617-3627 (2019).

39) UniProt C: UniProt: a worldwide hub of protein knowledge. *Nucleic Acids Res* **47,** D506-D515 (2019).

40) Altschul, S.F., Gish, W., Miller, W., Myers, E.W., Lipman, D.J. Basic local alignment search tool. *J Mol Biol* **215,** 403-410 (1990).

41) Domazet-Lošo, T. *et al*. No evidence for phylostratigraphic bias impacting inferences on patterns of gene emergence and evolution. *Mol. Bio. Evol.* **34,** 843-856 (2017).

42) Moyers, B.A., Zhang, J. Toward reducing phylostratigraphic errors and biases. *Genome Biol. Evol.* **10,** 2037-2048 (2018).

43) Anders, S., Huber, W. Differential expression analysis for sequence count data. *Genome Biology* **11,** R106 (2010).

44) Love, M.I., Huber, W., Anders, S. Moderated estimation of fold change and dispersion for RNA-seq data with DESeq2. *Genome Biol.* **15,** 550 (2014).

45) Alexa, A., Rahnenführer, J. topGO: Enrichment analysis for gene ontology. R package version 2.28.0. CRAN (2016).

46) Supek, F., Bošnjak, M., Škunca, N., Šmuc T. REVIGO summarizes and visualizes long lists of gene ontology terms. *PLOS One* **6,** e21800 (2011).

47) Dunrinck, S., Spellman, P.T., Birney, E., Huber, W. Mapping identifiers for the integration of genomic datasets with the R/Bioconductor package biomaRt. *Nature Protocols* **4,** 1184-1191 (2009).

48) Sinha, S., Liang, Y., Siggia, E. Stubb: a program for discovery and analysis of cis-regulatory modules. *Nucleic Acids. Res.* **34,** W555-W559 (2006).

49) Ament, S.A. *et al*. New meta-analysis tools reveal common transcriptional regulatory basis for multiple determinants of behavior. *PNAS* **109,** E1801-E1810 (2012).

50) Khan, A. *et al*. JASPAR 2018: update of the open-access database of transcription factor binding profiles and its web framework. *Nucleic Acids Res.* **46,** D260-D266 (2018).

SUPPLEMENTARY FIGURES

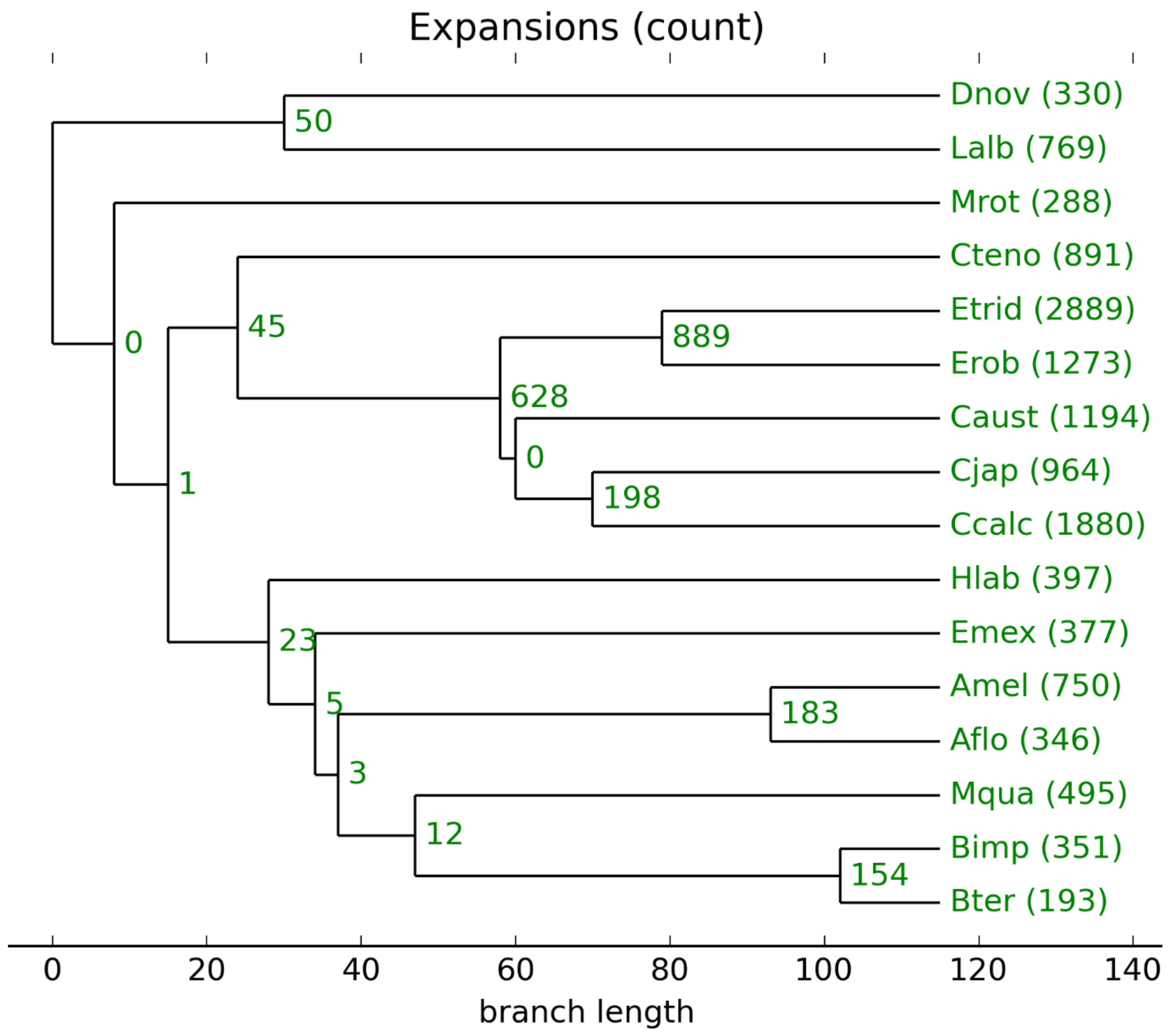

Figure S1. Total counts of gene family expansions by lineage determined by CAFE analysis; includes both significant and non-significant changes in gene family size.

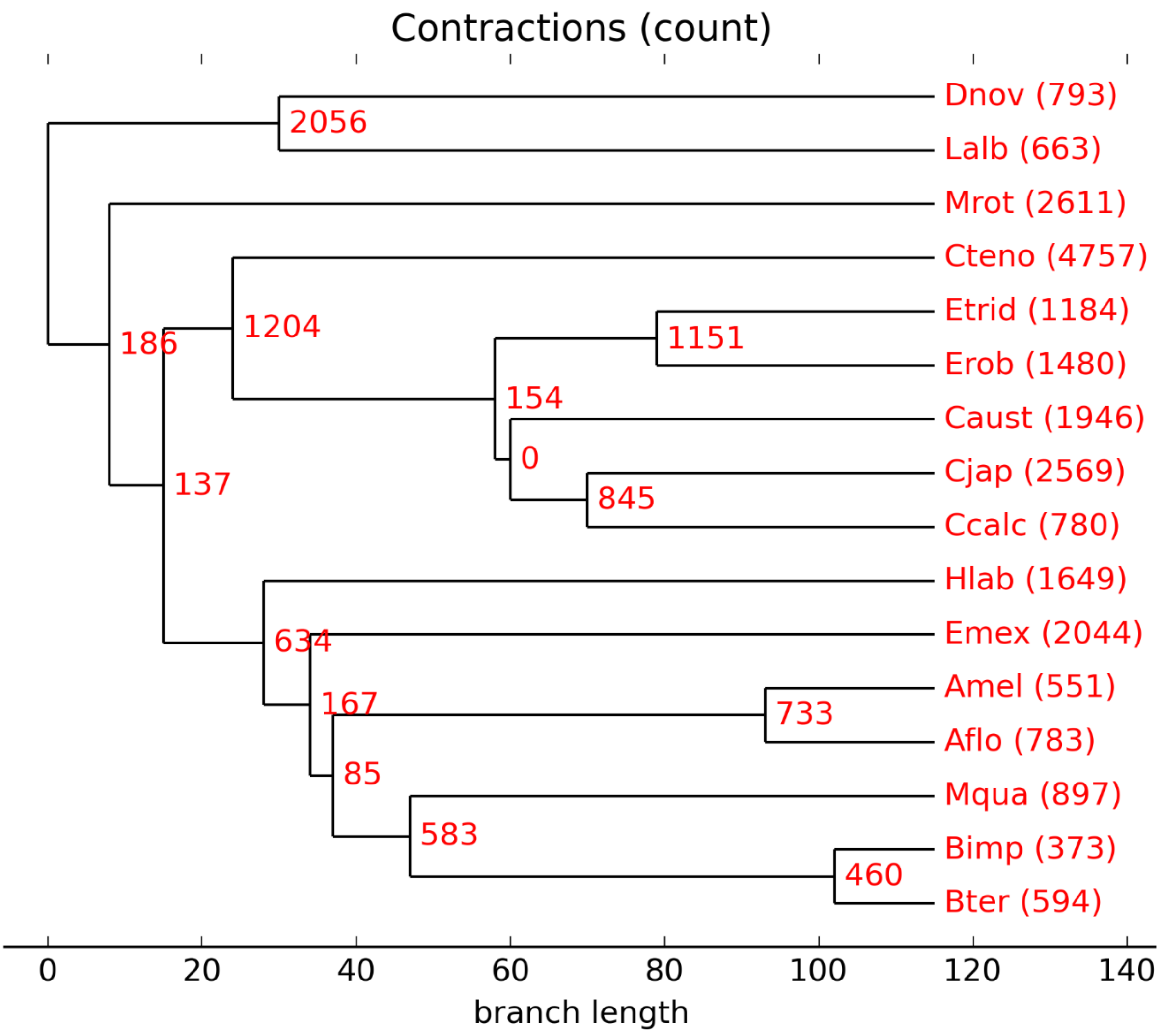

Figure S2. Total counts of gene family contractions by lineage determined by CAFE analysis; includes both significant and non-significant changes in gene family size.

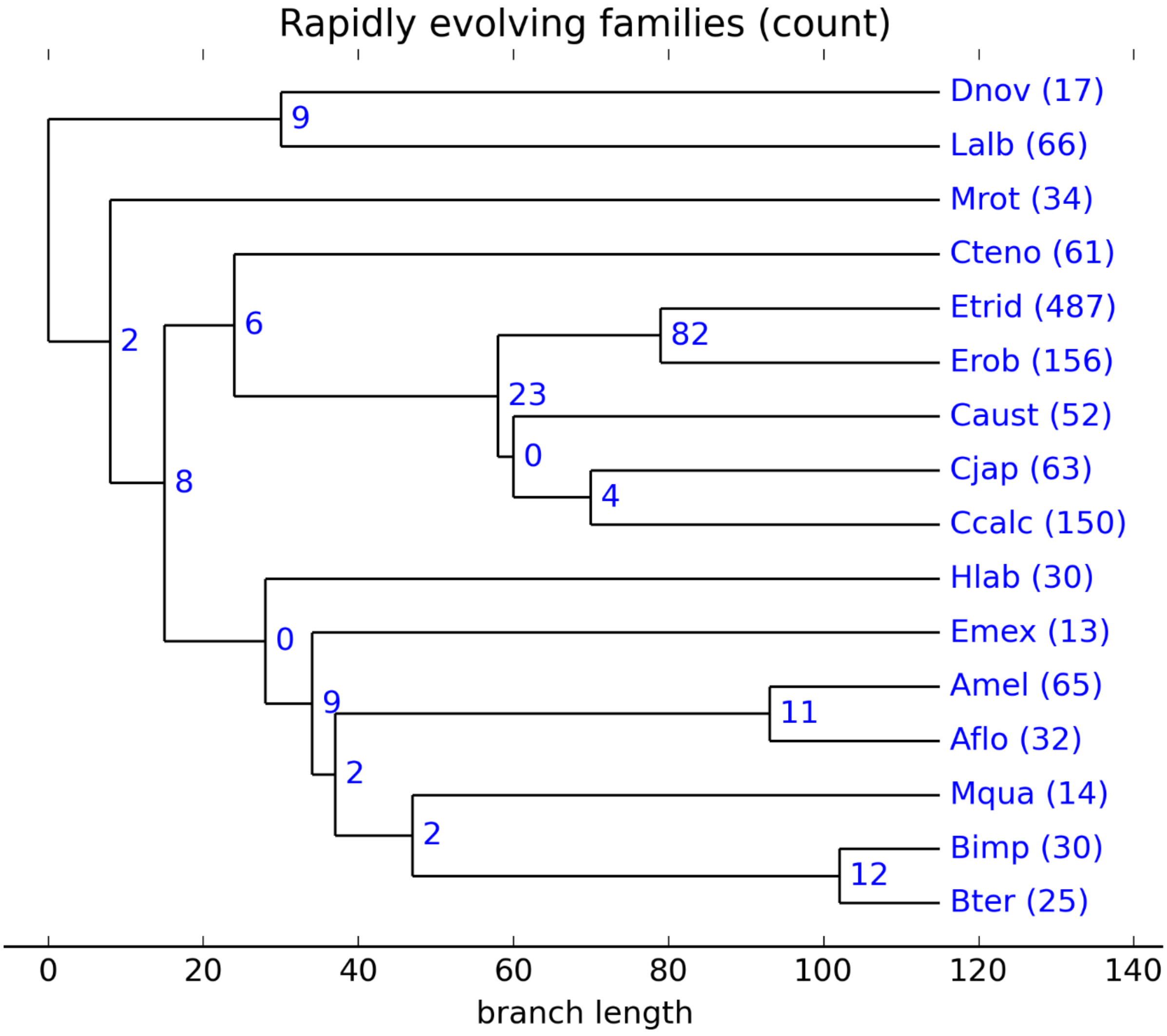

Figure S3. Total counts of rapidly evolving gene families by lineage determined by CAFE analysis; includes both significant and non-significant changes in gene family size.

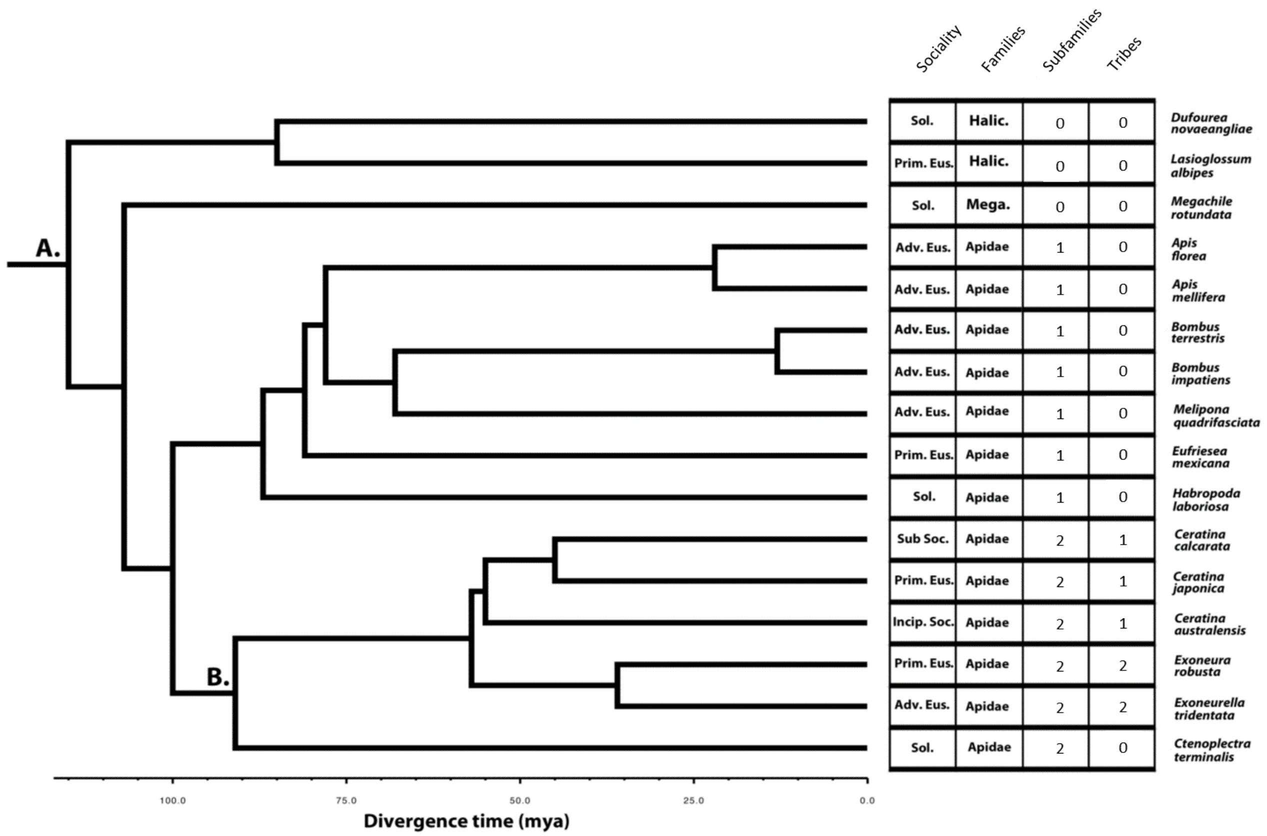

Figure S4. Full comparative study design as a phylogeny with trait mapping. Independent origins of sociality are indicated at A) and B); individual lineage sociality (Sol – solitary, Sub Soc – subsocial, Incip Soc – incipiently social, Prim Eus – primitively eusocial, and Adv Eus – advanced eusocial), family (Apidae, Halic – Halictidae, Mega – Megachilidae), subfamily (0 – outgroup, 1 – Apinae, 2 – Xylocopinae), and tribe (0 – outgroup, 1 – Ceratinini, 2 – Allodapini) membership status are then indicated. These social and phylogenetic bins were then used to structure Computational Analysis of gene Family Evolution (CAFE) and Phylogenetic Analysis by Maximum Likelihood (PAML) runs.

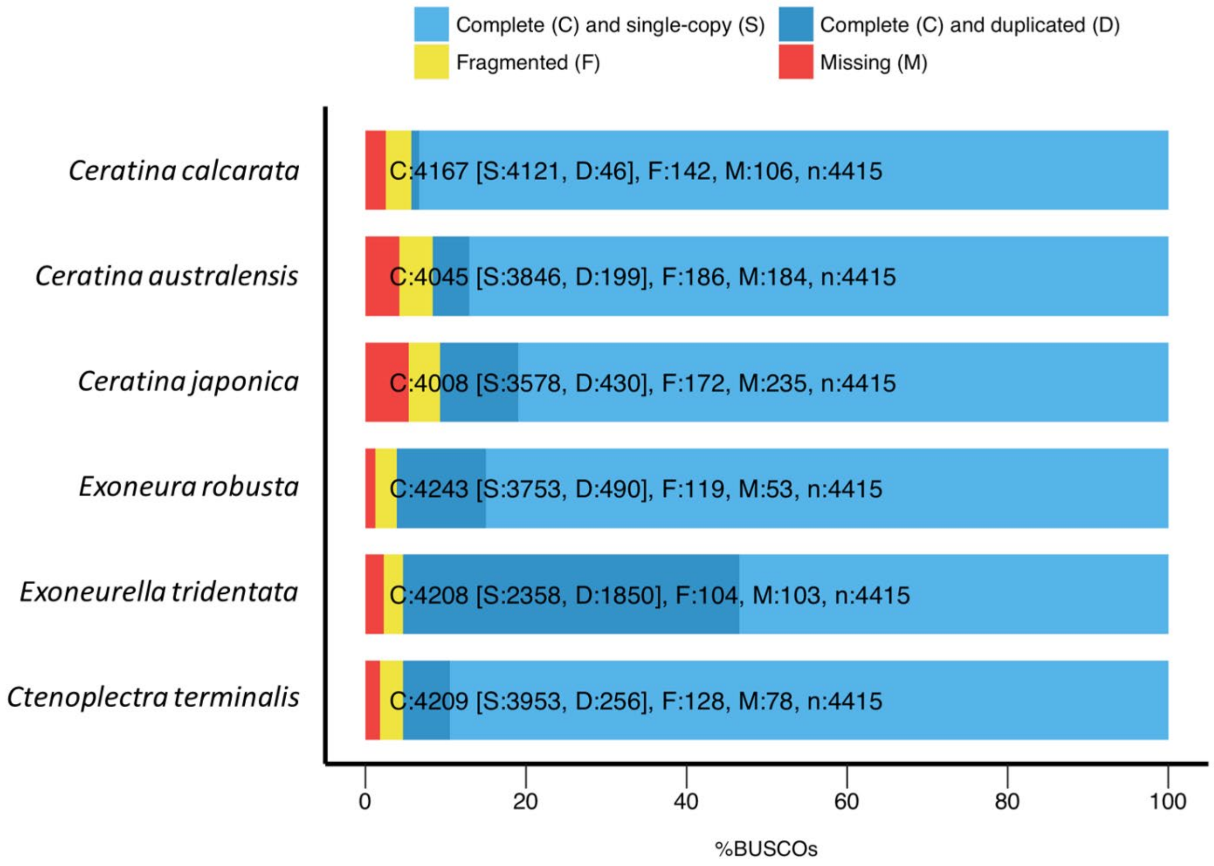

Figure S5. Results of BUSCO analysis for each of the newly for previously sequenced carpenter bee genomes (*C. calcarata*, Rehan et al. 2016; and *C. australensis* Rehan et al. 2018) along with each of the four genomes assembled *de novo* for the current study.

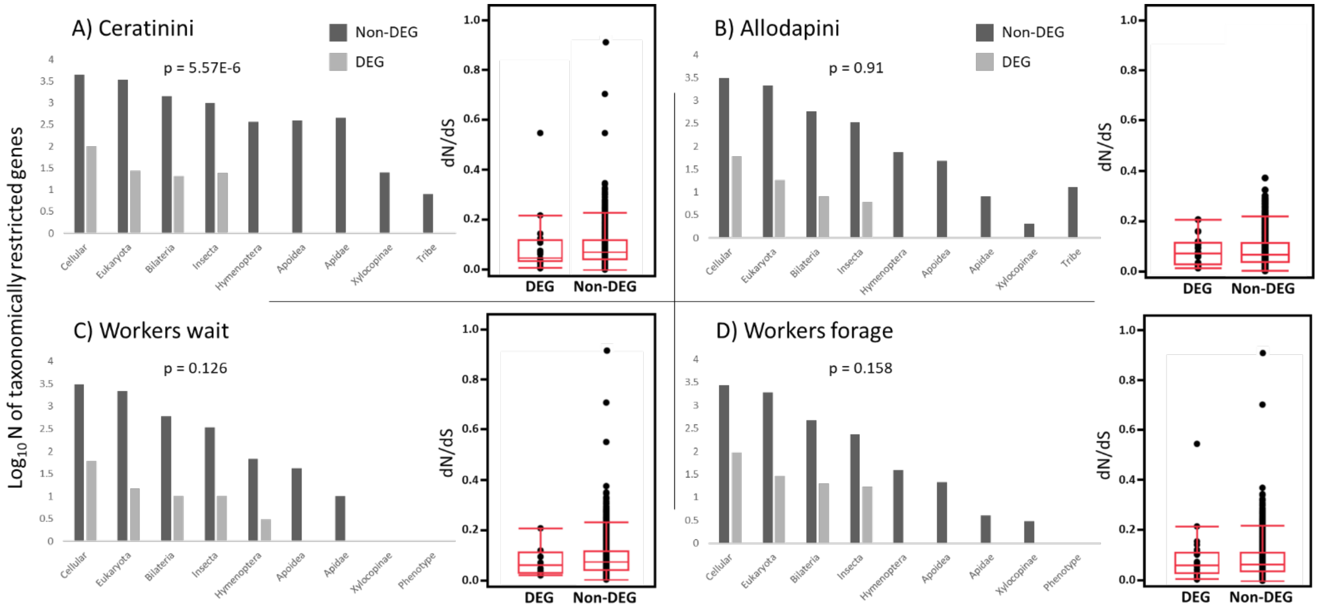

Figure S6. Distribution of differentially (DEG) and non-differentially expressed genes (Non-DEG) across several phylostratigraphic levels for A) Ceratinini (*C. japonica*, *C. australensis*, and *C. calcarata*), B) Allodapini (*E. robusta* and *E. tridentata*), C) xylocopine species in which workers wait (*C. australensis* and *E. robusta*), and D) xylocopine species in which workers forage (*C. japonica* and *E. tridentata*). DEGs are better represented among ancient conserved genes relative to non-DEGs in all groups, but significantly overrepresented among ancient genes only in Ceratinini (Cellular to Insecta vs Hymenoptera to Tribe; $\chi^2_{Ceratinini} = 20.63$, df=1, p = 5.57E-6; see Data S19). Included beside each distribution is a comparison of average dN/dS values between differentially and non-differentially expressed genes among each group. DEGs and non-DEGs feature similar (i.e. not significantly different) rates of protein evolution across all groups (e.g. Ceratinini; Wilcoxon Test, Z = -0.971, P = 0.3314; see Data S42).

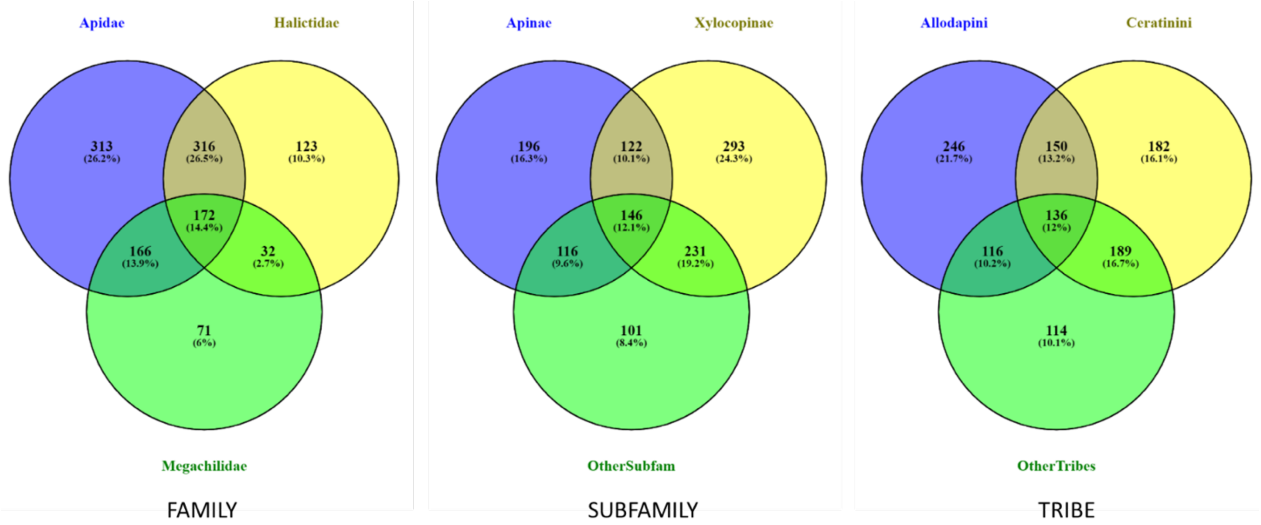

Figure S7. Side-by-side comparisons of orthogroups under significant positive selection based on different levels of phylogenetic comparison. From left to right, results of PAML analysis by Family, Subfamily, and Tribe. Apidae, Xylocopinae, and Allodapini each contain the largest numbers of genes under positive selection for their respective comparative brackets.

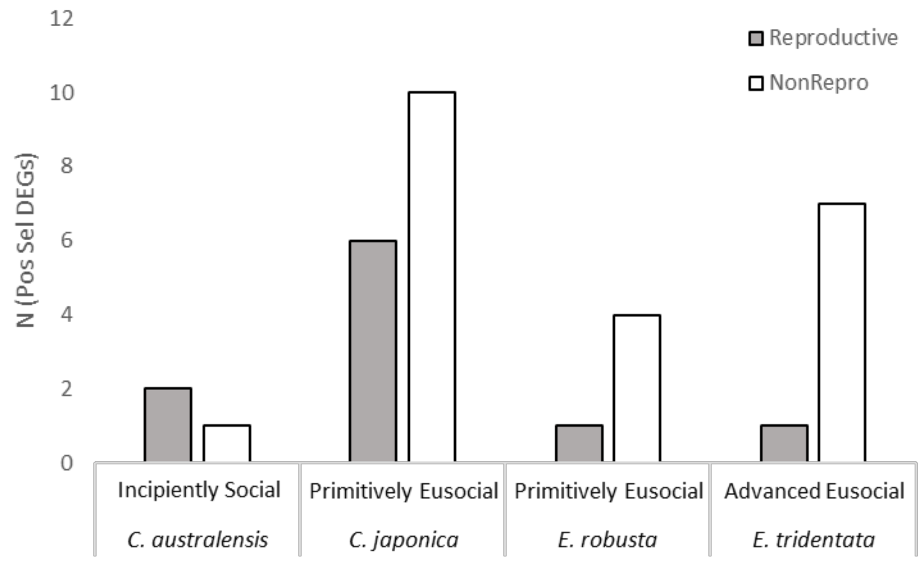

Figure S8. Bar graph displaying counts of differentially expressed genes identified among xylocopine species undergoing significant positive selection based on results of PAML. Overall, the ratio of non-reproductive to reproductive DEGs under positive selection increases with derivations in social complexity from incipiently social to advanced eusocial.

A

**Comparative differential gene expression**	*Exoneura robusta*		*Euglossa dilemma*			*Polistes metricus*	
Annotation of homologous genes	Queen	Worker	Foundress	Dominant	Subordinant	Queen	Worker
Histone H4							
Innexin inx7							
Protein mesh							
Endocuticle structural glycoprotein SgAbd-1							
Cytochrome P450 4g15							
Dipeptidyl aminopeptidase-like protein 6							
Titin							

B

**Comparative differential gene expression**	*Ceratina japonica*		*Euglossa dilemma*			*Polistes metricus*	
Annotation of homologous genes	Queen	Worker	Foundress	Dominant	Subordinant	Queen	Worker
Protein unc-45 homolog B							
Transferrin							
Heat shock 70 kDa protein cognate 4							
Cadherin-89D							
Cuticlin-1							

C

**Comparative differential gene expression**	*Ceratina australensis*		*Ceratina calcarata*			
Annotation of homologous genes	Social Primary	Social Secondary	Autumn Mother	Autumn Daughter	Foragers	Guards
bone morphogenetic protein 7-like						
sodium-dependent dopamine transporter						
carboxylesterase 5a-like						
1-phosphatidylinositol- -bisphosphate phosphodiesterase-like						
neurol calcium sensor 2						
fibrillin-2-like						
glucosamine--fructose-6-phosphate aminotransferase						
mical-like protein 2						
sodium potassium-transporting atpase subunit beta-2-like						
sodium- and chloride-dependent glycine transporter 2-like						
sodium- and chloride-dependent gaba transporter 1						

Figure S9. Tables illustrating homologous overlap of differentially expressed genes identified in the primitive eusocial caste systems of a) *E. robusta* vs *Euglossa dilemma* and *Polistes metricus*; and b) *C. japonica* vs *Euglossa dilemma* and *Polistes metricus*; and c) incipiently social *C. australensis* vs *Ceratina calcarata* . Reds and blues indicate gene up- and downregulation respectively; whites indicate a lack of homology. Patterns of differential gene expression are best conserved by phylogeny, with consistencies in both reproductive role and foraging/guarding status among similar social forms.
