# Supplementary material for: Sociality sculpts similar patterns of molecular evolution in two independently evolved lineages of eusocial bees
Source: Commun Biol. 2021 Feb 26;4:253. doi: 10.1038/s42003-021-01770-6 (PMC7977082; doi:10.1038/s42003-021-01770-6)
Supplement: Supplementary file 2 — Description of Additional Supplementary Files [file 42003_2021_1770_MOESM2_ESM.pdf]

Description of Additional Supplementary Files

File Name: Supplementary Data 1

Description:

Data S1 (Ginfo). de novo genome sample and accession data.

Data S2 (Gsize). Summary information on 16 bee genomes generated or accessed for comparative analyses.

Data S3 (Gstat). *de novo* genome assembly statistics.

Data S4 (Raw CAFE data). Raw data from CAFE analyses of gene family expansions and contractions. Orthogroup ID is indicated, followed by gene family counts in each lineage/group (columns B - AE). Tree topology, family-wide and lineage or group-specific significance values are then provided (columns AF - BK). Significant lineage/group-level values ($p<0.05$) are highlighted in yellow.

Data S5 (Exp|Cons by Lineage). Significant gene family expansions and contractions by lineage. Orthogroups are listed for each lineage test, along with associated species-specific genes, protein family IDs, context (expansion/contraction), gene family domain and annotation.

Data S6 (Exp|Cons by ingroup). Significant gene family (Orthogroup) expansions and contractions among ingroup Xylocopine taxa. Results are provided for both the subfamily and tribe levels (LineageLevel; LevelID). Context, protein family domain (OrthoID), family (OrthoID2), and orthoID (OrthoMCL ID) are provided where available. Ceratina australensis gene IDs and annotations are provided where available for each Orthogroup (CaustGene; CaustAnno).

Data S7 (Exps by Sociality). Summary of significant gene family expansions by sociality. Expanded orthogroups (OG) are listed for each degree of Sociality tested, gene family Domain and Keyword annotation.

Data S8 (C.australensis). All gene data for *Ceratina australensis* genome, as either differentially expressed or under positive selection in current study, and relevant annotation if available. Relevant data are then presented for differentially expressed genes (H-L), genes under positive selection and associated context (i.e. PAML analysis; M-U), and gene families undergoing significant expansion or contraction (i.e. CAFE analysis; V,W)

Data S9 (C.japonica). All gene data for *Ceratina japonica* genome, including Phylostrata level, Gene ID, PAML Orthogroup ID, status as either differentially expressed or under positive selection in current study, and relevant annotation if available. Relevant data are then presented for differentially expressed genes (H-P), genes under positive selection and associated context (i.e. PAML analysis; Q-Y), and gene families undergoing significant expansion or contraction (i.e. CAFE analysis; Z, AA)

Data S10 (E.robusta). All gene data for *Exoneura robusta* genome, including Phylostrata level, Gene ID, PAML Orthogroup ID, status as either differentially expressed or under positive selection in current study, and relevant annotation if available. Relevant data are then presented for differentially

expressed genes (H-P), genes under positive selection and associated context (i.e. PAML analysis; Q-AC), and gene families undergoing significant expansion or contraction (i.e. CAFE analysis; AD, AE)

Data S11 (E.tridentata). All gene data for *Exoneura tridentata* genome, including Phylostrata level, Gene ID, PAML Orthogroup ID, status as either differentially expressed or under positive selection in current study, and relevant annotation if available. Relevant data are then presented for differentially expressed genes (H-P), genes under positive selection and associated context (i.e. PAML analysis; Q-AC), and gene families undergoing significant expansion or contraction (i.e. CAFE analysis; AD, AE)

Data S12 (GOTerms_Mergers). Full list of gene ontology (GO) terms enriched among differentially expressed genes in each of our four ingroup species. GO term ID, annotation (Term), Type, broad biological enrichment contexts (D-F), and species-specific contexts of enrichment (G-N) are provided. Results of comparative analysis involving 14 additional studies are then presented (O - BJ).

Data S13 (BLAST_Framework). Resources used for BLASTn analyses comparing to ingroup genomes (A-F); and Studies used for comparative analysis with ingroup for which positive matches were found. Table includes species and behavioral contexts for each study (J-P), along with indicators of data overlap overall and by species (i.e. hits to results of behavior or age-associated tests; and whether data overlap comprises DEGs, GO terms, and/or transcription factor binding site motifs; Q-AE).

Data S14 (CausDEGMergers). All differentially regulated *Ceratina australensis* genes identified during DESeq analyses, their annotations, expression values, and biological contexts in which they were identified are presented in columns A through H. Homologous gene lists from other species (supported by BLASTn analysis) are provided in columns I through AB. Biological contexts from additional studies in which some of these homologous genes were also determined as differentially expressed are then provided in further columns (AC-DT).

Data S15 (CjapDEGMergers). All differentially regulated *Ceratina japonica* genes identified during DESeq analyses, their annotations, expression values, and biological contexts in which they were identified are presented in columns A through L. Homologous gene lists from other species (supported by BLASTn analysis) are provided in columns M through AH. Biological contexts from additional studies in which some of these homologous genes were also determined as differentially expressed are then provided in further columns (AI-EA).

Data S16 (ErobDEGMergers). All differentially regulated *Exoneura robusta* genes identified during DESeq analyses, their annotations, expression values, and biological contexts in which they were identified are presented in columns A through L. Homologous gene lists from other species (supported by BLASTn analysis) are provided in columns M through AE. Biological contexts from additional studies in which some of these homologous genes were also determined as differentially expressed are then provided in further columns (AF-CX).

Data S17 (EtridDEGMergers). All differentially regulated *Exoneurella tridentata* genes identified during DESeq analyses, their annotations, expression values, and biological contexts in which they were identified are presented in columns A through L. Homologous gene lists from other species (supported by BLASTn analysis) are provided in columns M through AG. Biological contexts from

additional studies in which some of these homologous genes were also determined as differentially expressed are then provided in further columns (AH-CR).

Data S18 (Phylostrata). Phylostrata binning for orthogroups assessed in PAML analysis. Condensed (A-E) and detailed phylostrata assignments are indicated (F-J). Sublevel phylostrata levels 19 and 20 is illustrated to right of table.

Data S19 (DEGPhylostata). X^2 calculations performed on differentially and non-differentially expressed genes, according to their phylostrata bins, among ingroup tribe (Ceratinini or Allodapini) and phenotype (workers wait or forage). In rows 3-57, counts of genes identified at each phylostrata level are provided (A-E) and chi square tables and calculations presented (H-R) for each group. Graphical interpretations of raw (U-AJ) and log transformed (AM-AY) count data are then presented.

Data S20 (PAML_OG_Contents). Orthogroup gene contents for all 16 species involved in analyses. For each orthogroup, genes are presented in a continuous, non-sorted row (up to AAC). Ascending orthogroup ID number coincides with descending orthogroup gene saturation.

Data S21 (PAML_OG_Core). Filtered list of 1631 orthogroups used in subsequent analyses of neutral and positive selection. Includes orthogroups which contain genes featuring a single copy for each species and which were detected in at least 10 of the 16 species. For each included orthogroup, Phylostrata (PS), Apis mellifera gene ID, NCBI I, Gene Symbol, annotation, and relevant key organism (Apis mellifera) are indicated

Data S22 (PAML Test Models). Models used for testing neutral and positive selection within phylogeny branches and sites. Test target category, name (Test), focal group, and phylogeny details are provided. "#1" coding in Details indicates focal groups of each test.

Data S23 (Apidae vs. Rest). Results of PAML analysis testing 1631 filtered orthogroups for evidence of positive selection focusing on Apidae family vs rest in 16 bee phylogeny. Results of branchwise testing (A-P) and sitewise (R-AC) testing are provided, including tested orthogroups, numbers of parameters (np), likelihood ratio test value (LRT), likelihood ratio (LR), degrees of freedom (df), critical value (CV), including background (b) and foreground (f) dN/dS values, tree length dN and dS valus, positive selection, phylostrata, and functional statuses.

Data S24 (Halictidae vs. Rest). Results of PAML analysis testing 1631 filtered orthogroups for evidence of positive selection focusing on Halictidae family vs rest in 16 bee phylogeny. Results of branchwise testing (A-P) and sitewise (R-AC) testing are provided, including tested orthogroups, numbers of parameters (np), likelihood ratio test value (LRT), likelihood ratio (LR), degrees of freedom (df), critical value (CV), including background (b) and foreground (f) dN/dS values, tree length dN and dS valus, positive selection, phylostrata, and functional statuses.

Data S25 (Mega. vs. Rest). Results of PAML analysis testing 1631 filtered orthogroups for evidence of positive selection focusing on Megachilidae family vs rest in 16 bee phylogeny. Results of branchwise testing (A-P) and sitewise (R-AC) testing are provided, including tested orthogroups, numbers of parameters (np), likelihood ratio test value (LRT), likelihood ratio (LR), degrees of freedom (df), critical value (CV), including background (b) and foreground (f) dN/dS values, tree length dN and dS valus, positive selection, phylostrata, and functional statuses.

Data S26 (Apinae vs. Rest). Results of PAML analysis testing 1631 filtered orthogroups for evidence of positive selection focusing on Apinae subfamily vs rest in 16 bee phylogeny. Results of branchwise testing (A-P) and sitewise (R-AC) testing are provided, including tested orthogroups, numbers of parameters (np), likelihood ratio test value (LRT), likelihood ratio (LR), degrees of freedom (df), critical value (CV), including background (b) and foreground (f) dN/dS values, tree length dN and dS valus, positive selection, phylostrata, and functional statuses.

Data S27 (Xylocopin. vs. Rest). Results of PAML analysis testing 1631 filtered orthogroups for evidence of positive selection focusing on Xylocopinae subfamily vs rest in 16 bee phylogeny. Results of branchwise testing (A-P) and sitewise (R-AC) testing are provided, including tested orthogroups, numbers of parameters (np), likelihood ratio test value (LRT), likelihood ratio (LR), degrees of freedom (df), critical value (CV), including background (b) and foreground (f) dN/dS values, tree length dN and dS valus, positive selection, phylostrata, and functional statuses.

Data S28 (SubfamOut vs. Rest). Results of PAML analysis testing 1631 filtered orthogroups for evidence of positive selection focusing on Outgroup subfamilies vs Apinae+Xylocopinae in 16 bee phylogeny. Results of branchwise testing (A-P) and sitewise (R-AC) testing are provided, including tested orthogroups, numbers of parameters (np), likelihood ratio test value (LRT), likelihood ratio (LR), degrees of freedom (df), critical value (CV), including background (b) and foreground (f) dN/dS values, tree length dN and dS valus, positive selection, phylostrata, and functional statuses.

Data S29 (Ceratinini vs. Rest). Results of PAML analysis testing 1631 filtered orthogroups for evidence of positive selection focusing on Ceratinini tribe vs rest in 16 bee phylogeny. Results of branchwise testing (A-P) and sitewise (R-AC) testing are provided, including tested orthogroups, numbers of parameters (np), likelihood ratio test value (LRT), likelihood ratio (LR), degrees of freedom (df), critical value (CV), including background (b) and foreground (f) dN/dS values, tree length dN and dS valus, positive selection, phylostrata, and functional statuses.

Data S30 (Allodapini vs. Rest). Results of PAML analysis testing 1631 filtered orthogroups for evidence of positive selection focusing on Allodapini tribe vs rest in 16 bee phylogeny. Results of branchwise testing (A-P) and sitewise (R-AC) testing are provided, including tested orthogroups, numbers of parameters (np), likelihood ratio test value (LRT), likelihood ratio (LR), degrees of freedom (df), critical value (CV), including background (b) and foreground (f) dN/dS values, tree length dN and dS valus, positive selection, phylostrata, and functional statuses.

Data S31 (TribesOut vs. Rest). Results of PAML analysis testing 1631 filtered orthogroups for evidence of positive selection focusing on outgroup tribes vs Ceratinini + Allodapini in 16 bee phylogeny. Results of branchwise testing (A-P) and sitewise (R-AC) testing are provided, including tested orthogroups, numbers of parameters (np), likelihood ratio test value (LRT), likelihood ratio (LR), degrees of freedom (df), critical value (CV), including background (b) and foreground (f) dN/dS values, tree length dN and dS valus, positive selection, phylostrata, and functional statuses.

Data S32 (Sol vs. Rest). Results of PAML analysis testing 1631 filtered orthogroups for evidence of positive selection focusing on lineages of solitary sociality vs rest in 16 bee phylogeny. Results of branchwise testing (A-P) and sitewise (R-AC) testing are provided, including tested orthogroups, numbers of parameters (np), likelihood ratio test value (LRT), likelihood ratio (LR), degrees of freedom (df), critical value (CV), including background (b) and foreground (f) dN/dS values, tree length dN and dS valus, positive selection, phylostrata, and functional statuses.

Data S33 (SubSoc vs. Rest). Results of PAML analysis testing 1631 filtered orthogroups for evidence of positive selection focusing on lineage of subsociality vs rest in 16 bee phylogeny. Results of branchwise testing (A-P) and sitewise (R-AC) testing are provided, including tested orthogroups, numbers of parameters (np), likelihood ratio test value (LRT), likelihood ratio (LR), degrees of freedom (df), critical value (CV), including background (b) and foreground (f) dN/dS values, tree length dN and dS valus, positive selection, phylostrata, and functional statuses.

Data S34 (IncpSoc vs. Rest). Results of PAML analysis testing 1631 filtered orthogroups for evidence of positive selection focusing on lineages of incipient sociality vs rest in 16 bee phylogeny. Results of branchwise testing (A-P) and sitewise (R-AC) testing are provided. Tested orthogroups, numbers of parameters (np), likelihood ratio test value (LRT), likelihood ratio (LR), degrees of freedom (df), critical value (CV), including background (b) and foreground (f) dN/dS values, tree length dN and dS valus, positive selection, phylostrata, and functional statuses are all provided.

Data S35 (PrimEus vs. Rest). Results of PAML analysis testing 1631 filtered orthogroups for evidence of positive selection focusing on lineages of primitive eusociality vs rest in 16 bee phylogeny. Results of branchwise testing (A-P) and sitewise (R-AC) testing are provided, including tested orthogroups, numbers of parameters (np), likelihood ratio test value (LRT), likelihood ratio (LR), degrees of freedom (df), critical value (CV), including background (b) and foreground (f) dN/dS values, tree length dN and dS valus, positive selection, phylostrata, and functional statuses.

Data S36 (AdvEus vs. Rest). Results of PAML analysis testing 1631 filtered orthogroups for evidence of positive selection focusing on lineages of advanced eusociality vs rest in 16 bee phylogeny. Results of branchwise testing (A-P) and sitewise (R-AC) testing are provided, including tested orthogroups, numbers of parameters (np), likelihood ratio test value (LRT), likelihood ratio (LR), degrees of freedom (df), critical value (CV), including background (b) and foreground (f) dN/dS values, tree length dN and dS valus, positive selection, phylostrata, and functional statuses.

Data S37 (Early vs. Rest). Results of PAML analysis testing 1631 filtered orthogroups for evidence of positive selection focusing on lineages of early sociality (i.e. solitary, subsocial, incipiently social) vs rest in 16 bee phylogeny. Results of branchwise testing (A-P) and sitewise (R-AC) testing are provided, including tested orthogroups, numbers of parameters (np), likelihood ratio test value (LRT), likelihood ratio (LR), degrees of freedom (df), critical value (CV), including background (b) and foreground (f) dN/dS values, tree length dN and dS valus, positive selection, phylostrata, and functional statuses.

Data S38 (Late vs. Rest). Results of PAML analysis testing 1631 filtered orthogroups for evidence of positive selection focusing on lineages of late sociality (i.e. primitive and advanced eusocial) vs rest in 16 bee phylogeny. Results of branchwise testing (A-P) and sitewise (R-AC) testing are provided, including tested orthogroups, numbers of parameters (np), likelihood ratio test value (LRT), likelihood ratio (LR), degrees of freedom (df), critical value (CV), including background (b) and foreground (f) dN/dS values, tree length dN and dS valus, positive selection, phylostrata, and functional statuses.

Data S39 (ChiSquare). Chi square test paramters and statistics comparing orthogroups under positive selection and not under positive selection among all social phenotypes, between Early and Late stage social bins; at the family and tribal levels; and among differentially expressed genes (DEGs) associated with reproductive or non-reproductive roles in each of the ingroup taxa

Data S40 (GO_by_Selection). GO term enrichment among orthogroups found to be under significant positive selection during PAML. Gene ontological terms and GO enrichment are based on single-copy A. mellifera data as used during orthogroup assignment. Study level and context columns are provided for quick sorting; GO ID, annotation (term), type, and results of Fisher's exact tests (TopGO) are presented.

Data S41 (GO_by_Phenotype). GO term enrichment among ingroup species as assessed by shared phenotype. GO ID, annotation (term), type, and biological contexts (reproductive status and worker phenotype) are presented.

Data S42 (TFBSmotifs_Mergers). TFBS motif enrichment identified by cis-Metalysis analysis for all differentially regulated genes among all ingroup species. Reference database, motif cobination (for paired motifs), functional motif, primary and secondary motif IDs, full name of motif 1, their biological contexts of enrichment and regulatory directions in this study are all defined in columns A through Q. Overlaps with additional studies are then provided in columns R through X.

Data S43 (ChiSquare Tests). Results of Chi-Square tests comparing counts of upregulated DEGs vs enriched TFBS motifs by reproductive status, lineage, and phenotype among xylocopine species.

Data S44 (WilcoxonTests_A). Results of Wilcoxon (Mann-Whitney) tests comparing dN/dS scores of DEGs vs non-DEGs under positive selection. No tests were found to be significant

Data S45 (WilcoxonTests_B). Wilcoxon test statistics used to compare dN/dS values among phylogenetic and phenotypic categories; significant test results are indicated in bold
